# scGGC: a two-stage strategy for single-cell clustering through cellular gene pathway construction

**DOI:** 10.1093/bib/bbaf368

**Published:** 2025-07-23

**Authors:** Zhi Zhang, Qiucheng Sun, Chunyan Wang, Songrun Jiang

**Affiliations:** College of Computer Science and Technology, Changchun Normal University, Changchun 130032, China; College of Computer Science and Technology, Changchun Normal University, Changchun 130032, China; College of Computer Science and Technology, Changchun Normal University, Changchun 130032, China; College of Computer Science and Technology, Changchun Normal University, Changchun 130032, China

**Keywords:** scRNA-seq, dimensionality reduction, cell-gene interaction, generative adversarial networks, high-confidence cells

## Abstract

In the last few years, there has been great advancement in the field of single-cell data investigation, particularly in the development of clustering methods. The advanced research is increased for the development of clustering algorithms tailored for single-cell RNA sequencing data. Conventional methods primarily focus on local relationships among cells or genes, while overlooking the global cell-gene interactions. As a result, the high dimensionality, noise, and sparsity of the data continue to pose significant challenges to clustering accuracy. To address the challenges of single-cell clustering analysis, we propose a novel single-cell clustering model, scGGC, which integrates graph autoencoders and generative adversarial network techniques. The innovations of scGGC include two components: (i) construction of an adjacency matrix that incorporates cell–cell and cell-gene relationships to capture complex interactions in a graph structure, enabling nonlinear dimensionality reduction and initial clustering via a graph autoencoder; (ii) enhancement of clustering performance by selecting high-confidence samples from the initial clusters for adversarial neural network training. A comprehensive evaluation on nine publicly available scRNA-seq datasets demonstrates that scGGC outperforms eight comparison methods. For example, on datasets such as MHC3K, the Adjusted Rand Index increases by an average of 10.1%. Furthermore, marker gene identification and cell type annotation further confirm the biological relevance of scGGC, with marker gene overlap rates exceeding 70% across multiple datasets. We conclude that scGGC not only improves the accuracy of single-cell data clustering but also enhances the identification of cell-type-specific marker genes. The scGGC code is available at https://github.com/Zhi1002/scGGC.

## Introduction

Single-cell RNA sequencing (scRNA-seq) is a transformative technology for resolving cellular heterogeneity, offering high-resolution insights into gene expression at the single-cell level [1]. It has advanced research in immunology and oncology by elucidating regulatory networks and key signaling pathways [2–4]. Despite these achievements, clustering cell subpopulations from sparse and noisy scRNA-seq data remains a major challenge [5].

Bioinformatics and various tools of bioinformatics play important role in the prediction of proteins targeting drugs discovery and drugs development. Different studies have been demonstrated the implication of various tools [6–8]. These predictors involved in advance research on various diseases including cancer including lung cancer [9–11]. In this context, numerous clustering approaches have been developed to better uncover the intricate structural features inherent in scRNA-seq data. Traditional distance-based clustering methods such as K-means effectively achieve cell grouping on low-dimensional data by minimizing the Euclidean distance [12]. In addition, hierarchical clustering reveals hierarchical relationships among cells by progressively merging or partitioning cell populations to form a dendrogram [13]. However, as the complexity of scRNA-seq datasets increases, these methods have limited performance in capturing complex interrelationships between cells. Graph theory-based clustering methods have emerged in this context, such as the Louvain algorithm in Seurat, which reveals community features in the data through shared nearest neighbor graphs [14]. Based on Louvain’s idea, the SOUP method further implements soft clustering through similarity matrices to identify pure cells and transition-state cells [15]. To ensure stable performance amid noise and complexity, integrated clustering methods have been gradually developed. For example, RaceID [16] and CIDR [17] further enhance robustness through specialized techniques. Nevertheless, these approaches often fall short in capturing non-linear dependencies and the high-dimensional nature of scRNA-seq data.

Deep neural networks, with their hierarchical structures and nonlinear mapping capabilities, have shown strong performance in feature extraction and are widely used in single-cell clustering analysis [18]. Deep Embedded Clustering utilizes an autoencoder to generate a low-dimensional representation and combines it with K-means for external clustering. Furthermore, scMCs [19] integrate single-cell transcriptomic and epigenetic data, employing attention mechanisms and contrastive learning strategies to extract both unique and shared attributes from multi-omics datasets. With the wide application of graph neural networks in bioinformatics, some approaches incorporate cell–cell graphs or gene-cell graphs into the encoder [20], significantly enhancing the ability to model cell-gene relationship networks (Supplementary Fig. S1–S2). By constructing inter-cellular graph structures and combining graph embedding techniques with graph convolutional networks, Graph-sc [21] enhances the modelling capability of intercellular relationships. scGGAN [22] builds gene relationship networks and combines GCN with generative adversarial networks (GANs) to address missing values in scRNA-seq data while enhancing clustering performance and preventing overfitting. In addition, scMAE [23] efficiently integrates inter-gene interactions through multi-attribute graph convolutional networks to more comprehensively model cell type-specific gene expression patterns. To reduce reliance on external clustering, adaptive encoders like scDeepCluster [24] embed the clustering loss within the encoder by generating pseudo-labels and iteratively optimizing the clustering results. To effectively capture the structural features of high-dimensional data, the SCVI model [25] introduces variational inference to model cellular features in the latent space and achieves clustering by learning the latent distribution of cellular expression [26]. Given the increasing demand for accuracy, semi-supervised methods that combine a small amount of labeled data with large-scale unlabeled data are gaining traction. To make full use of the label information, AttentionAE-sc [27] dynamically adjusts the feature weights through the attention mechanism to enhance the clustering effect. scTPC leverages ternary loss and pairwise constraints to construct structured relationships among labeled cells [28]. The accuracy of labels directly impacts model generalization, making the generation of high-quality training labels a key challenge for optimizing clustering performance.

To this end, we propose scGGC, a semi-supervised clustering method based on GANs. First, we construct the overall neighborhood matrix by integrating cell-gene interaction information to more comprehensively reflect the complex interactions between cells and genes. Based on this, the graph autoencoder is employed to reduce dimensionality and capture the complex nonlinear structure of the data. The resulting low-dimensional latent variables are then preliminarily clustered using K-means. Based on the preliminary clustering results, the distance of each cell to the center of mass within its cluster is calculated, and the cell closest to the center of mass is selected as a high-confidence sample to more accurately represent the structural characteristics of the cluster. Using the obtained high-confidence samples, the GAN model is trained to optimize the clustering results again, thus improving the generalization ability and accuracy of the model. In contrast to Graph-sc and scGGAN, scGGC innovatively constructs a unified cell-gene adjacency matrix to model multi-level interactions, and employs adversarial training guided by high-confidence samples to refine clustering results. The phased design of the method allows us to effectively deal with the high-dimensional nature and complexity of single-cell data, significantly improving the accuracy and biological explanatory power of clustering. In the next section, we present the implementation process and technical details of the method.

## Methods

### Data preprocessing

**Figure 1:**
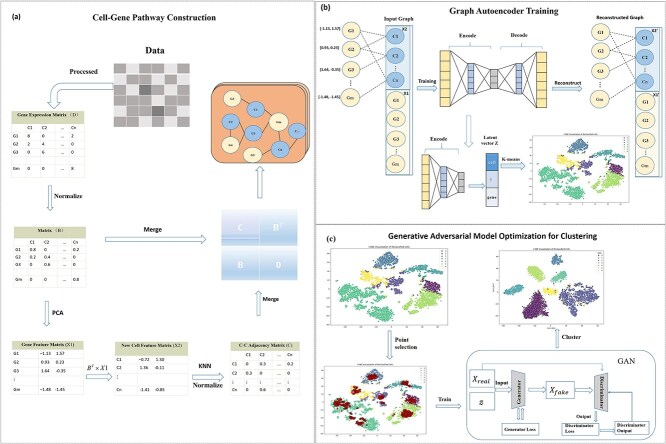
Workflow of the scGGC. The scGGC method consists of three core modules: (a) cell-gene pathway construction. (b) graph autoencoder training. (c) adversarial network embedding optimization.

The overall framework of scGGC is shown in Fig. 1. To guarantee the quality of data and the accuracy of analysis, we preprocess the raw gene expression data. Specifically, the gene expression matrix is $\mathrm{DF}\in{\mathrm{R}}^{\mathrm{m}\times \mathrm{n}}$, where m is the number of genes and n is the number of cells. First, we remove genes with nonzero expression in <1% of cells. This step eliminates lowly expressed and noisy genes, retaining only biologically relevant ones. Then, we calculate the variance of each gene in the dataset and selected the 2000 genes with the highest variance as the feature gene expression matrix ${\mathrm{D}}^{\prime}\in{\mathrm{R}}^{2000\times \mathrm{n}}$. Finally, we perform standardization and normalization on the processed gene expression data to obtain the matrix $\mathrm{D}$.

### Cell-gene pathway construction

scGGC applies column-wise normalization to the preprocessed gene expression matrix $\mathrm{D}$, resulting in a normalized matrix $\mathrm{B}\in{\mathrm{R}}^{\mathrm{m}\times \mathrm{n}}$. In $\mathrm{B}$, the element $\mathrm{B}\left[\mathrm{i},\mathrm{j}\right]$ represents the normalized expression level of the i-th gene in the j-th cell. Simultaneously, this can be interpreted as the relevance measure between the j-th cell and i-th gene. Consequently, the matrix $\mathrm{B}$ can be viewed as an adjacency matrix describing the relationships between cells and genes. Subsequently, via principal component analysis (PCA), each gene row in matrix $\mathrm{B}$ is reduced to h dimensions (Supplementary Fig. S3), generating a feature expression matrix representing genes, denoted as $\mathrm{X}1$. Using matrix $\mathrm{B}$, we further derived a new feature expression matrix $\mathrm{X}2$ for cells. Based on the gene expression coefficients from the gene expression matrix, the expression profile of each cell is computed as a linear combination of gene expression levels. The calculation is formulated as follows:


(1) \begin{equation*} \mathrm{X}2={\mathrm{B}}^{\mathrm{T}}\cdot \mathrm{X}1 \end{equation*}

The inter-cell distance matrix $\mathrm{P}$ is constructed by calculating the Euclidean distance between each cell in the reduced feature matrix $\mathrm{X}2$. Based on the matrix $\mathrm{P}$, the k-nearest neighbors (KNN) algorithm is applied to connect each cell to its KNN. Subsequently, the rows of the resulting matrix are normalized, thereby forming a sparse adjacency matrix $\mathrm{C}$ representing the connections between cells.

In molecular regulatory networks, genes play an important role in regulating cellular characteristics. Conversely, the cellular environment influences gene function by modulating gene expression, thereby forming a complex bidirectional feedback mechanism. To comprehensively capture the multidimensional interactions between cells and genes, we construct the complete adjacency matrix $\mathrm{A}$ in the following form:


(2) \begin{equation*} {\displaystyle \begin{array}{@{}cc@{}}& \mathrm{n}\kern4.75em m\\{}\begin{array}{@{}c@{}}n\\{}m\end{array}& \left[\begin{array}{@{}cc@{}}\lambda \cdotp C& \left(1-\lambda \right)\cdotp{B}^T\\{}\left(1-\lambda \right)\cdotp B& 0\end{array}\right]\end{array}}=A \end{equation*}

Where n is the number of cells, m is the number of genes,and 0 is an $\mathrm{m}\times \mathrm{m}$ zero matrix, $\lambda \in \left(0,1\right)$ is used to adjust the difference between data, detailed example see (Supplementary Fig. S4).

### Graph autoencoder training

In order to effectively retain the structural information of the data, scGGC employs a graph autoencoder model for dimensionality reduction. The complete adjacency matrix $\mathrm{A}$ is used as the graph structure, combined with the feature information of the nodes $\mathrm{X}=\left[\mathrm{X}2;\mathrm{X}1\right]\in{\mathrm{R}}^{\left(\mathrm{m}+\mathrm{n}\right)\times \mathrm{h}}$ and passed into the graph self-encoder. The encoder [29] progressively maps the input features to the low-dimensional space through multi-layer graph convolution to extract the higher-order feature representations, and at the same time optimizes the node features using the graph structure information. The computational process of the graph convolution layer is defined as follows:


(3) \begin{equation*} {X}^{\left(l+1\right)}= BatchNorm\left( LeakyRely\left(A\cdot{X}^{(l)}{W}^{(l)}\right)\right) \end{equation*}

where ${\mathrm{X}}^{\left(\mathrm{l}\right)}$ is the node feature matrix of the l-th layer, ${W}^{(l)}\in{R}^{h\times d}$ is the learnable weight matrix, and d is the output feature dimension. We combine the node feature matrix with the adjacency matrix A to aggregate information from neighboring nodes, and then update features using the weight matrix. The output feature matrix ${X}^{\left(l+1\right)}$ transforms in dimension to $\left(\mathrm{m}+\mathrm{n}\right)\times \mathrm{d}$, thereby progressively extracting higher-order feature representations.

After the graph convolution operation, the final graph embedding vector is used to represent the low-dimensional features of the node, denoted as $\mathrm{Z}={\mathrm{X}}_{\mathrm{final}}$. In the decoder, the low-dimensional embedding representation Z is processed through multilayer linear transformation combined with nonlinear activations to generate the feature representation for reconstruction. Subsequently, the decoder converts the decoded feature representation into node reconstruction results through an inner product operation, which is defined as follows:


(4) \begin{equation*} \hat{\mathrm{A}}=\mathrm{\sigma} \left(\mathrm{Z}{\mathrm{Z}}^{\mathrm{T}}\right) \end{equation*}

Where $\hat{\mathrm{A}}$ is the reconstructed adjacency matrix and $\mathrm{\sigma}$ is the activation function (such as Sigmoid), which is used to compress the output value within the [0,1] range.

To quantify the difference between the reconstructed adjacency matrix and the original adjacency matrix, we introduce the reconstruction residuals as the optimization objective, expressed as follows:


(5) \begin{equation*} {\mathcal{L}}_{recon}={\left\Vert A-\hat{A}\right\Vert}_F \end{equation*}

where, ${\left\Vert \cdot \right\Vert}_F$ represents the Frobenius norm.

To improve scalability and generalization on large-scale datasets, we incorporate a regularization term into the loss function as an extension (see Supplementary Fig. S5). After completing the model training, we extract the front n rows of the embedding vector $\mathrm{Z}$, the corresponding embeddings ${\mathrm{Z}}_{\mathrm{cells}}\in{\mathrm{R}}^{\mathrm{n}\times \mathrm{d}}$, where n is the number of cells and d is the dimension of the embedding space. Then, ${\mathrm{Z}}_{\mathrm{cells}}$ is clustered by the K-Means to generate preliminary cluster labels.

### Embedding optimization for generative adversarial networks

Based on the preliminary clustering results, we calculate the Euclidean distance of all points in each cluster to its center of mass and select the points in close proximity to the center of mass as high-confidence samples ${\mathrm{x}}_{\mathrm{real}}=\left\{{\mathrm{x}}_{1},{\mathrm{x}}_2,\dots, {\mathrm{x}}_{\mathrm{i}}\right\}$, ${\mathrm{x}}_i$ represents the collection of selected cell samples in each cluster. It is recommended that points in the range of the first 30%–50% of the distance from the center of mass be selected from each cluster as training samples. For details on high-confidence sample selection, refer to Supplementary Fig. S6.

The generator network architecture of scGGC consists of three fully connected layers. Initially, the noise vector $\mathcal{z}\in{R}^d$ and high-confidence cell features ${\mathrm{x}}_{\mathrm{real}}$ are concatenated and fed into the first layer, which maps the input to a 1024-dimensional space. Subsequently, dimensionality is progressively reduced via 512-dimensional and 256-dimensional fully connected layers, culminating in the generation of pseudo-cell features that match the dimensionality of genuine cell features. Each layer is followed by a LeakyReLU activation function with a negative slope of 0.2. The final layer employs a Tanh activation function to constrain the output within the range of [−1,1]. The generation process of the pseudo-sample $\hat{\mathrm{x}}$ is expressed as:


(6) \begin{equation*} \hat{\mathrm{x}}=\hat{G}\left({\mathrm{x}}_{\mathrm{real}},\mathcal{z}\right)= tach\left({\mathcal{W}}_g\cdot \left[{\mathrm{x}}_{\mathrm{real}}\Big\Vert \mathcal{z}\right]+{b}_g\right) \end{equation*}

where, ${\mathcal{W}}_{\mathrm{g}}$ and ${\mathrm{b}}_{\mathrm{g}}$ are the weight matrix and bias terms of the generator, respectively, $\left[{\mathrm{x}}_{\mathrm{real}}\Big\Vert \mathcal{z}\right]\in{\mathrm{R}}^{\mathrm{k}+\mathrm{d}}$, the Tanh activation function limits the output to the range of $\left[-1,1\right]$.

During the training process, the fake samples $\hat{\mathrm{x}}$ are fed into the discriminator along with the real sample ${\mathrm{x}}_{real}$, and the discriminator optimizes itself by distinguishing between the two. The loss function of the discriminator comprises both adversarial loss and classification loss, which is as follows:


(7) \begin{align*} {L}_D=&-{\mathbb{E}}_{\mathrm{x}\sim{\mathrm{p}}_{\mathrm{data}}\left(\mathrm{x}\right)}\left[\log \hat{D}(x)\right]-{\mathbb{E}}_{\mathcal{z}\sim{\mathrm{p}}_{\mathcal{z}}\left(\mathcal{z}\right)}\left[\log \Big(1-\hat{D}\left(\hat{G}\left(\mathcal{z}\right)\right)\right]\nonumber \\ &+{\mathbb{E}}_{\mathrm{x}\sim{\mathrm{p}}_{\mathrm{data}}\left(\mathrm{x}\right)}\left[\mathrm{CE}\Big({\hat{D}}_{\mathrm{CLS}}\left(\mathrm{x}\right),{y}_{real}\right] \end{align*}

where ${\mathrm{p}}_{\mathrm{data}}\left(\mathrm{x}\right)$ denotes the true data distribution, $\hat{\mathrm{D}}\left(\mathrm{x}\right)$ is the probability that the discriminator will judge a sample ${\mathrm{x}}_{\mathrm{real}}$ as true, $\hat{\mathrm{G}}\left(\mathcal{z}\right)$ is the sample generated by the generator, ${\mathrm{p}}_{\mathcal{z}}\left(\mathcal{z}\right)$is the noise distribution of the generator input, ${\hat{\mathrm{D}}}_{\mathrm{CLS}}$ is used for predicting the category of a sample, and ${\mathrm{y}}_{\mathrm{real}}$ corresponds to the labels of real sample. The discriminator is optimized by maximizing the probability of correctly identifying real samples while minimizing the probability of incorrectly classifying generated samples.

The generator optimizes the output by minimizing the loss function to reduce the discriminator into not being able to distinguish the generated samples from the real ones. As training progresses, the generator continuously improves its generated samples to better approximate the characteristics of the real samples, while the discriminator’s ability to distinguish them also improves. The loss function of the generator is defined as:


(8) \begin{equation*} {L}_G=-{\mathbb{E}}_{\mathrm{z}\sim{\mathrm{p}}_{\mathcal{z}}\left(\mathcal{z}\right)}\left[\log \Big(\hat{D}\left(\hat{G}\left(\mathcal{z}\right)\right)\right]+{\mathrm{E}}_{\mathrm{z}\sim{\mathrm{p}}_{\mathrm{z}}\left(\mathrm{z}\right)}\left[\mathrm{CE}\Big({\hat{\mathrm{D}}}_{\mathrm{CLS}}\left(\hat{\mathrm{G}}\left(\mathrm{z}\right)\right),{\mathrm{y}}_{\mathrm{fake}}\right] \end{equation*}

where, ${\mathrm{y}}_{\mathrm{fake}}$ represents the target class labels for the generated samples.

The generator and discriminator are both trained using the Adam optimizer, which adaptively tunes the learning rate by estimating the first and second moments of gradients, thereby improving optimization efficiency. To mitigate overfitting in the later stages of training, a learning rate scheduler is applied, halving the learning rate every five epochs.

After the training, we use the discriminator to re-categorize the cells to get the class probability of each cell. Assume that the features of all cells are represented as a matrix $\mathrm{T}\in{\mathrm{R}}^{\mathrm{k}\times \mathrm{d}}$, where k is the number of samples and d is the feature dimension of each sample. The output matrix of the discriminator is $\hat{\mathrm{D}}\left(\mathrm{T}\right)$, $\mathrm{D}{\left({\mathrm{T}}_{\mathrm{i}}\right)}_{\mathrm{j}}$ denoting the discriminator’s probability that the i-th cell belongs to the category j. The $\hat{y_i}$ predicted label of the i-th cell is defined as:


(9) \begin{equation*} \hat{y_i}= argmaxD{\left({T}_i\right)}_j \end{equation*}

## Results

### Datasets and methods description

We used nine scRNA-seq datasets from different tissues, organs, species, and sequencing platforms (including 10x Genomics, National Center for Biotechnology Information, and European Molecular Biology Laboratory) as benchmark datasets to investigate the annotation performance of scGGC on real-world data. Table 1 provides a brief description of each real dataset, including cell number, genes, cell types, and cell origin.

**Table 1:** Content display of the real dataset.

Dataset	Cells	Genes	Cell type	Origin
pbmc4k	4340	33 694	7	10x Genomics
MKTA1K	1385	32 285	9	10x Genomics
Kasper	4351	33 694	9	10x Genomics
Shiokawa	4449	17 712	10	NCBI
Sun	6360	44 271	7	NCBI
Leary	1489	23 323	12	EMBL_EBI
Schyns	6666	19 870	8	EMBL_EBI
MHC3K	3282	32 285	8	10x Genomics
Efremova	2130	16 861	13	EMBL_EBI

Our comparative study covers eight benchmark algorithms, focusing on analyzing their performance in scRNA-seq data processing. For spatial reconstruction, we selected four algorithms with distinct clustering strategies, including Seurat with a nearest-neighbor clustering strategy; RaceID and SC3 [30] based on consensus clustering, and identification of pure cells by semi-soft clustering of SOUP. These algorithms are grounded in traditional techniques such as graph theory and provide diverse ways to process scRNA-seq data. In the field of deep learning, we evaluate four models, including scDSC [31] based on deep semi-supervised clustering; Graph-sc, which uses a graph autoencoder to generate embeddings; CellVGAE [32], which based on variational graph autoencoder, and scMAE, which uses a combination of a masking mechanism and a self-encoder. Each of these approaches proposes different technical solutions to cope with problems specific to scRNA-seq data (such as high dimensions, sparsity, and dropout events), covering a wide range of approaches from graph theoretic modeling to deep learning.

We compared these algorithms with scGGC using appropriately configured parameter settings. The experimental framework evaluated their performance through a series of metrics including clustering accuracy, computational efficiency, and the ability to identify biologically significant cell subtypes. The subsequent sections present a detailed comparison of the results, highlighting the strengths and limitations of each method.

### Real data assessment

In this section, in order to explore the plausibility of potential features generated by different methods, we tested the effect of nine clustering methods using the nine real scRNA-seq datasets mentioned in Section Datasets and methods description, with the goal of demonstrating the effectiveness of scGGC.

**Figure 2:**
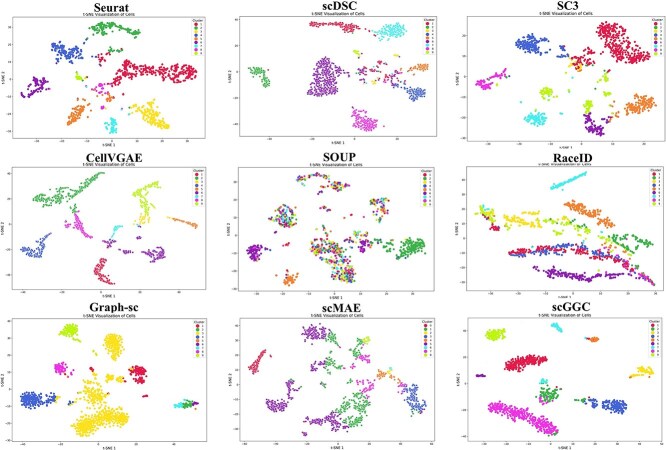
Cell distribution and clustering performance of scGGC.

To visually evaluate the performance of each clustering method, we applied t-SNE [33] to project the data into a 2D space for visualization. Using the MKTA1K dataset as an example, it was established that nuclei from the kidneys of adult C57/Bl6 mice are classified into nine distinct cell subtypes. As shown in Fig. 2, the scGGC model demonstrated a high degree of clustering separation and clearly distinguished different cell populations. CellVGAE and scDSC demonstrated superior performance in preserving the spatial structure between cells by employing graph embedding and diffusion-based spatial correlation methods. In contrast, Seurat and Graph-sc exhibited poor overall separation, with significant overlap between clusters. Additionally, SC3 and scMAE tended to divide the same cell cluster into multiple sub-clusters. SOUP and RaceID also performed poorly in clustering, failing to form a clear cluster structure, with significant overlap between cells. The corresponding visualization results for the remaining datasets are presented in Supplementary Fig. S7–S14.

**Figure 3:**
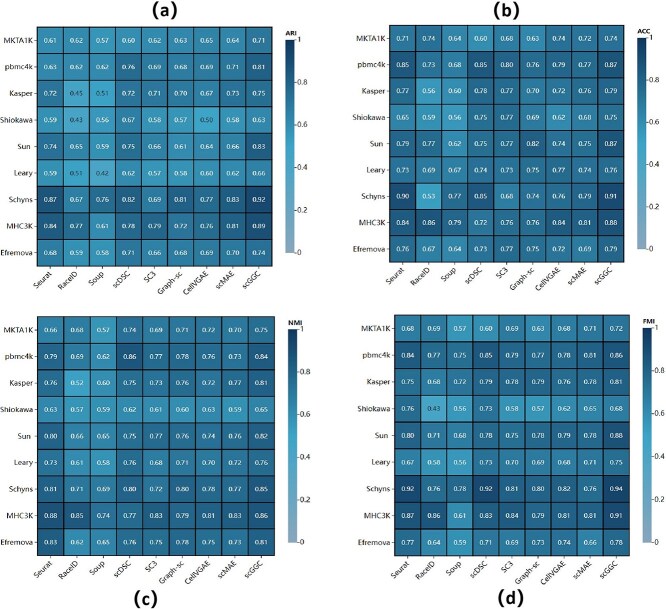
Clustering performance evaluation by (a) ARI. (b) ACC. (c) NMI. (d) FMI.

To quantitatively assess the performance of different clustering algorithms across multiple datasets (Fig. 3), we used three evaluation metrics: the Adjusted Rand Index (ARI) [34], Accuracy (ACC) [35], Normalized Mutual Information (NMI) [36], and the Fowlkes–Mallows Index (FMI) [37]. The detailed formulas for these calculations can be found in the Supplementary Materials. As shown in Fig. 3a, scGGC achieved the highest ARI score on most of the datasets. For example, on the Schyns [38] dataset, scGGC achieved an ARI of 0.92, which was 12.1% higher than that of the scDSC method. Figure 3b demonstrated the clustering effect of scGGC based on the ACC metrics, especially in the MHC3K dataset, where, compared to the Graph-sc method, scGGC improved by 15.7%. Similarly, Fig. 3c presents the evaluation results of NMI. For example, on the Kasper dataset, scGGC achieves an NMI of 0.81, which is superior to other methods. In addition, the FMI results shown in Fig. 3d further demonstrated the performance advantage of scGGC, which performs well in the majority of datasets.

**Figure 4:**
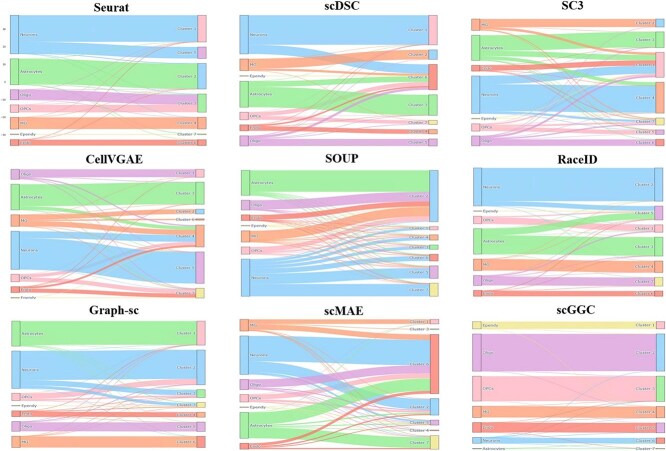
Sankey plot comparing clustering results of scGGC with other methods.

We used Sankey plots to visualize more directly the correspondence between the clustering results of each method and the real cell types. Using the Sun [39] dataset (Fig. 4) as an example, scGGC basically realizes the delineation of each type effectively. Although some cell types (e.g. Astrocytes and Neurons) were somewhat dispersed among different clusters, scGGC was still able to categorize most of the cells into the best clusters. In contrast, methods such as Seurat, SC3, scMAE, and CellVGAE grouped multiple cell types into the same cluster during the clustering process, leading to a decrease in classification accuracy. In addition, the clustering results of methods such as RaceID, Graph-sc, and SOUP exhibited poor alignment with real cell types. Overall, the high-precision matching further demonstrates the potential and robustness of scGGC in accurately classifying different cell types.

### Identification of auxiliary marker genes

**Figure 5:**
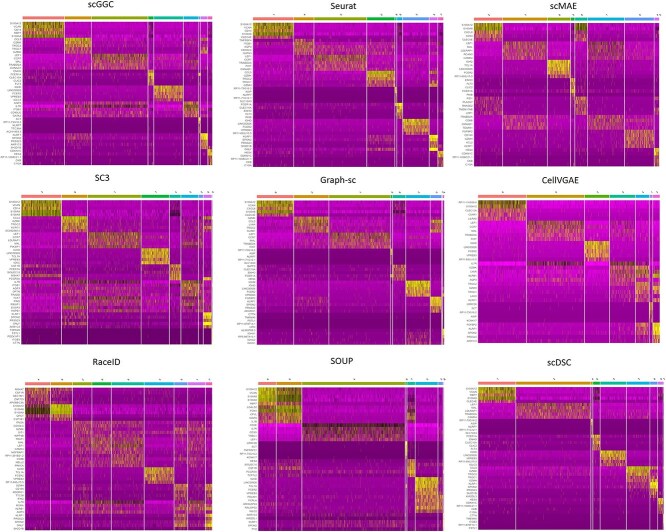
Heatmap of marker gene distribution for scGGC and other clustering methods.

In this section, we use the FindAllMarkers function in the Seurat package to identify the marker genes of each group based on the clustering labels. Figure 5 shows the marker gene distribution patterns of each subgroup obtained by the nine clustering methods in the dataset Kasper, where the top five marker genes are selected in each cluster. The clear demarcation of scGGC clusters and the distinct distribution of high and low-expression regions of the genes indicated strong discriminatory ability and stability, especially in the second and last clusters where the differences were significant. In contrast the heatmaps of RaceID and CellVGAE showed overlapping gene expression among multiple clusters, indicating a low level of differentiation. In addition, the gene expression patterns of other methods such as Seurat, SC3, Graph-sc, and scMAE showed some overall dispersion and ambiguity, although they formed relatively concentrated regions of high expression in some clusters. The corresponding visualization results of the rest of the datasets are in Supplementary Fig. S15–S16. Combined with the clustering distribution plots and metrics analyses in Section Real data assessment, scGGC demonstrates its clustering accuracy on both mathematical and biological levels.

To establish a connection between the computational clustering results and their biological significance, we performed systematic cell type annotation of the Schyns dataset using the ImmGen reference database. Supplementary Fig. S18(a) present a t-SNE visualization based on scGGC clustering results, where different clusters exhibit clear spatial separation in the low-dimensional space. Further cell type annotation shown in Supplementary Fig. S18(b) reveals that Cluster 0 (purple) is highly enriched in macrophages, whereas Cluster 7 (red) exhibits a strong match with the expression profile of the urothelial marker gene Upk3b. Notably, the spatial expression patterns of marker genes illustrated in Supplementary Fig. S19 are highly consistent with the annotation results, further supporting the reliability and accuracy of our method in identifying cell types. The expression patterns of relevant marker genes and corresponding annotation results are detailed in the Supplementary materials.

Based on the annotation framework described above, we selected the top 100 marker genes from each cluster. These marker genes were manually matched with the publicly available marker genes in CellMark, a cellular marker database [40]. For each cluster, the corresponding overlap rate was calculated. Subsequently, the average overlap rate across all clusters was computed to provide a comprehensive evaluation of the model’s performance.

**Figure 6:**
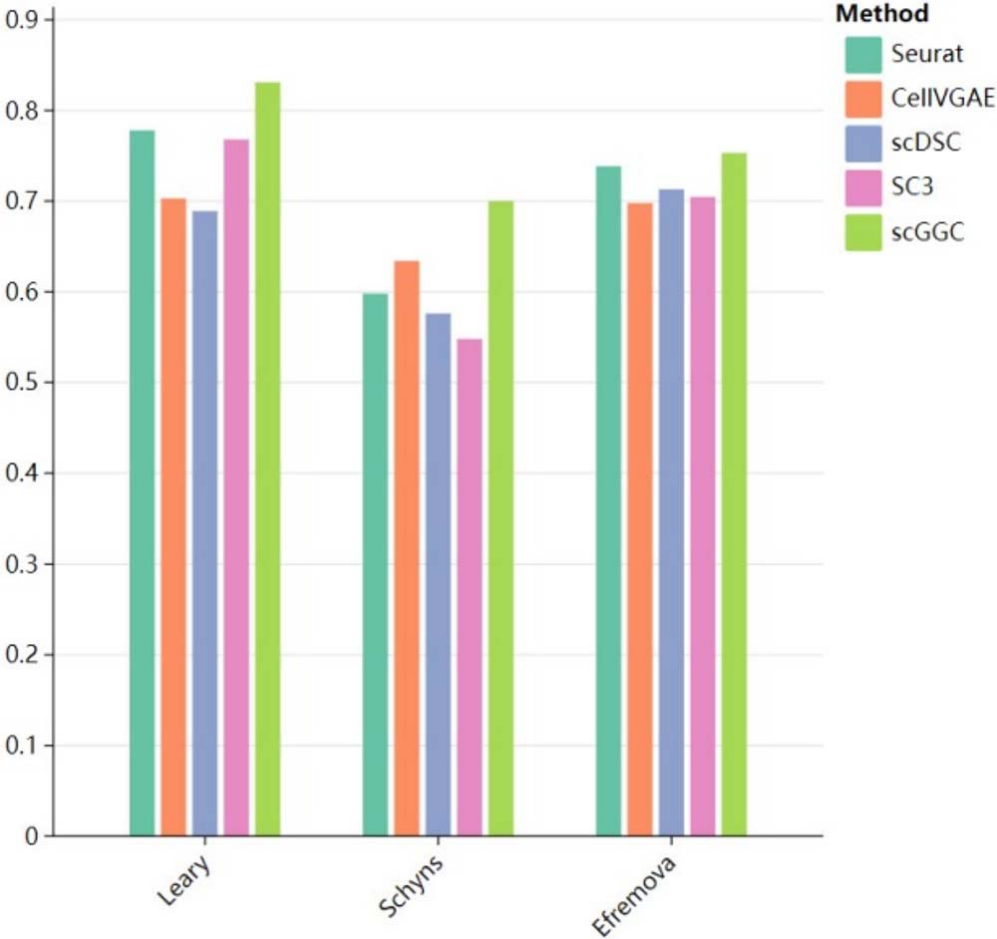
Comparative analysis of marker gene overlap rates across datasets.

Taking the Leary [41], Schyns, and Efremova [42] datasets as an example, Fig. 6 illustrated the average overlap rate of scGGC and the other four methods (Seurat, CellVGAE, scDSC, SC3), and the specific overlap rate data for each cluster can be found in Supplementary Fig. S20–S22. The results demonstrate that scGGC exhibits a high degree of overlap across all datasets, with an average overlap exceeding 0.8 in the Leary dataset, indicating its superiority in marker gene identification. The scDSC method performs relatively consistently across all datasets, with the average overlap rate remaining stable around 0.7. SC3 and Seurat display moderate performance, with overlap rates slightly lower than those of scGGC. In contrast, CellVGAE consistently achieves lower overlap rates across all datasets. By analyzing the overlap rate of marker genes, we further validated the ability of scGGC to identify specific cell subgroups in high-dimensional scRNA-seq data.

### Bidirectional regulation and optimization of cell-gene pathways

**Figure 7:**
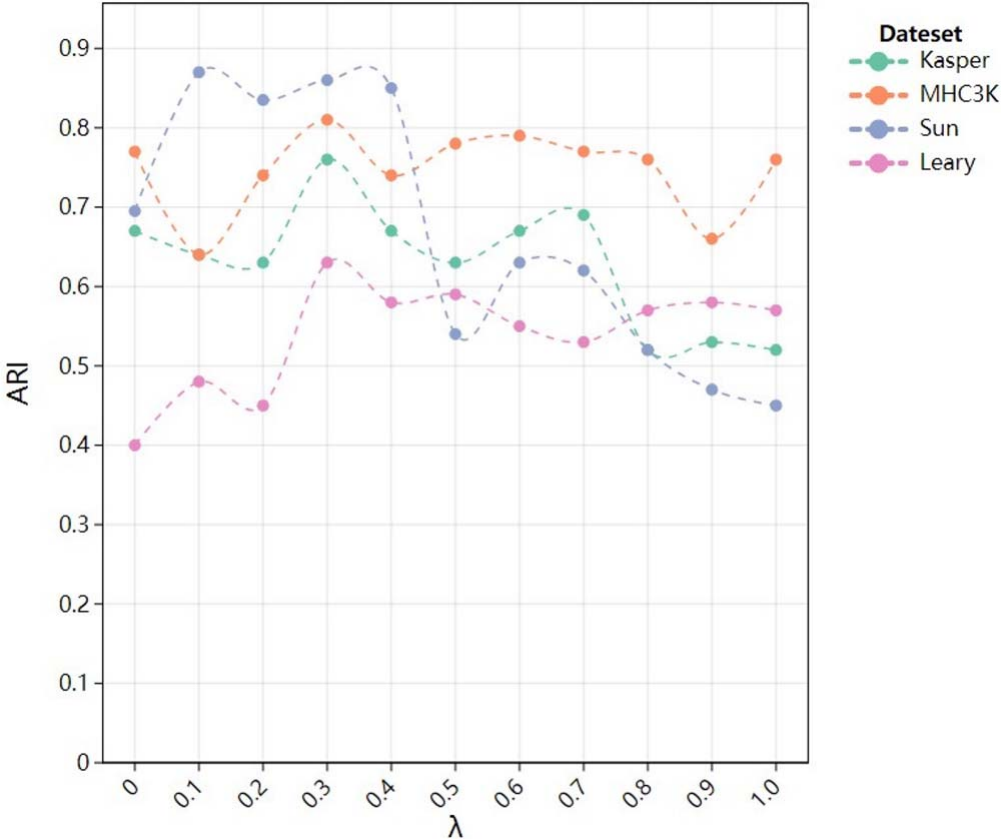
Model comparison in capturing cell-gene interaction relationships.

The relationship between cells and genes is complex and interdependent, with bidirectional interactions influencing both. We iteratively tuned the parameter $\mathrm{\lambda}$ several times to capture the interactions between cells and genes. In this experiment, we gradually increased the value $\mathrm{\lambda}$ from 0.1 to 1.0 and observed its effect on the model performance. As shown in Fig. 7, for the MHC3K dataset, the model reached the highest value of ARI (close to 0.8) at values $\mathrm{\lambda}$ between 0.2 and 0.4, indicating that the model is better able to capture important cell-gene interactions at this point while effectively avoiding disturbances caused by overfitting. Similarly, the ARI for the Kasper and Sun datasets performed relatively well over the same range, but exhibited slightly higher volatility. On the other hand, in the Leary dataset, the model reached the highest ARI at lower values $\mathrm{\lambda}$ (0.3 to 0.4), followed by a decline in performance, showing that overfitting occurs earlier in this dataset as the connection strength increases.

The experimental results showed that the model performed best in capturing the complex relationships between cells and genes, with high clustering accuracy when the value $\mathrm{\lambda}$ lies between 0.2 and 0.4. This experimental result was consistent with the characterization of scRNA-seq data. scRNA-seq data are highly heterogeneous and contain complex cell-gene expression relationships. If the model overly relies on cell-to-cell relationships, important cell-gene expression patterns may be overlooked, leading to loss of information. With our approach, the model comprehensively considers the bidirectional relationships between cells and genes, and achieve a reasonable balance in the characterization of connection strengths. This enables the model to effectively capture key cell-gene interactions, enhancing its biological explanatory power and reliability.

### Embedding optimization for generative adversarial networks

**Figure 8:**
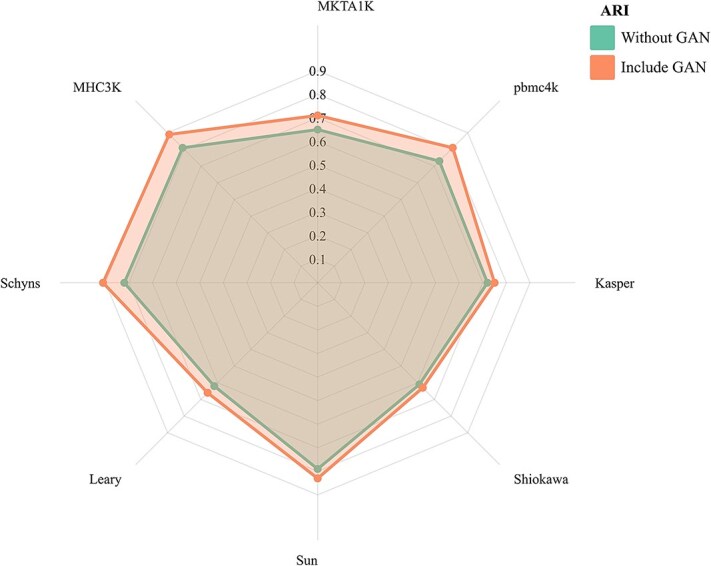
Optimization effect of GAN on clustering performance.

We perform ablation experiments to evaluate the effect of the GAN on the overall model clustering accuracy, and use the ARI to quantify the clustering results. As shown in Fig. 8, the adversarial model shows different degrees of performance improvement over multiple datasets (Leary, Schyns, MHC3K, Shiokawa [43], Sun, MKTA1K, etc.) compared to the no-adversarial model. In specific datasets, such as MHC3K and Schyns, the improvement with the addition of the GAN was particularly significant, indicating that the GAN contributed more to the model clustering ability when dealing with these datasets. In other datasets, such as pbmc4k and Kasper, the adversarial model still outperformed the base model, albeit with a smaller boost. Overall, the introduction of GAN improved the clustering accuracy by ~9.1% on average compared to the non-adversarial model.

The results of this experiment suggested that scGGC, through the adversarial mechanism of the generator and the discriminator, enabled the generator to learn the distribution of the data and generate virtual samples with similar characteristics to the real samples, capturing subtle differences that are not adequately represented in the initial clustering. Overall, GAN made a significant contribution to the model in the task of clustering single-cell datasets.

## Discussion and conclusion

In this study, we proposed a semi-supervised clustering model, scGGC, which integrates a graph self-encoder and a GAN to enhance the clustering of scRNA-seq data. The model constructs a comprehensive cell-gene neighbor-joining matrix and employs a two-stage clustering strategy to capture complex cell-gene interactions. Validation on multiple scRNA-seq datasets demonstrates that, under adversarial training guided by high-confidence samples, scGGC achieves an average clustering accuracy improvement of 9.1% compared to the first stage, significantly enhancing the recognition of true cell population structures. Moreover, scGGC further confirms its efficacy in single-cell clustering analysis by accurately identifying specific marker genes (such as Cldn5 and Upk3b). However, the performance of this method is highly dependent on hyperparameter tuning and initial conditions in the adversarial network module. Future work could introduce an adaptive hyperparameter adjustment method within scGGC. Furthermore, extending the integration to multi-omics data, particularly the joint analysis of transcriptomics, ATAC-seq, and proteomics data, will provide more comprehensive information for classifying cell populations. By integrating multiple data modalities, the model will not only reveal complex intracellular regulatory networks but also uncover changes in chromatin accessibility and protein expression, thus further refining the classification of cell subpopulations.

## Data sources

The link to the data from the unreferenced sources is as follows:

- The pbmc4k dataset from:

(https://support.10xgenomics.com/single-cell-gene-expression/datasets/2.1.0/pbmc4k)

- The MKTA1K dataset from:

(https://www.10xgenomics.com/datasets/1k-mouse-kidney-nuclei-isolated-with-chromium-nuclei-isolation-kit-3-1-standard)

- The Kasper dataset from:

(https://support.10xgenomics.com/single-cell-gene-expression/datasets/2.1.0/pbmc4k)

- The MHC3K dataset from:

(https://www.10xgenomics.com/datasets/5k-adult-mouse-heart-nuclei-isolated-with-chromium-nuclei-isolation-kit-3-1-standard)

**Key Points**

- We introduce a clustering model known as scGGC, which combines a Graph Self-Encoder with Generative Adversarial Networks (GANs), designed for the efficient clustering of single-cell RNA sequencing data.
- By constructing an integrated cell-gene adjacency matrix and modulating the weight coefficients between cells and between cells and genes, the scGGC model is capable of capturing the intricate interactions between cells and genes at a deeper level.
- Building upon the initial clustering, scGGC further incorporates an adversarial neural network. By calculating the distances from cells within each cluster to their centroids, high-confidence samples closest to the centroids are selected as training data. The final clustering results were optimized by using the adversarial network training.
- Experiments conducted across multiple single-cell RNA sequencing datasets demonstrate that the scGGC exhibits superior performance in terms of clustering accuracy and the identification of cell-specific marker genes.

## Supplementary Material

Supplementary_bbaf368(1)
