# Supplementary material for: scGGC: a two-stage strategy for single-cell clustering through cellular gene pathway construction
Source: Brief Bioinform. 2025 Jul 23;26(4):bbaf368. doi: 10.1093/bib/bbaf368 (PMC12284768; doi:10.1093/bib/bbaf368)
Supplement: Supplementary_bbaf368(1) [file supplementary_bbaf368(1).docx]

**Supplementary for scGGC**

1. **Cell–gene relational graph**


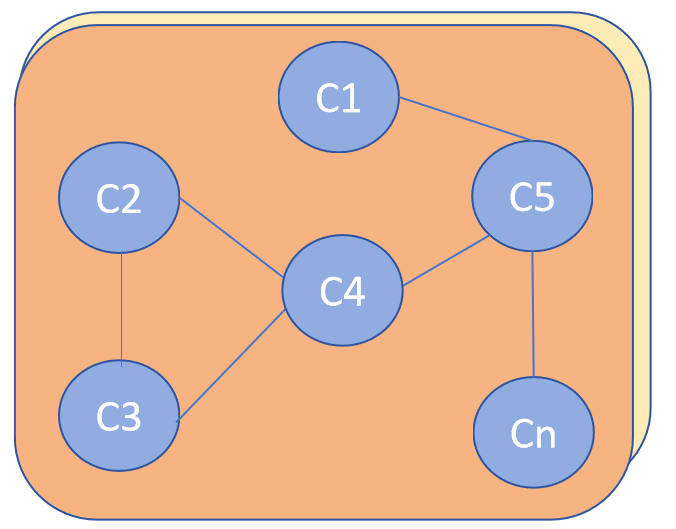


Figure S1. Modeling cells in scRNA-seq data using a graph structure. In the graph, nodes represent cells, and edges represent cell similarity. Each cell node is represented as a weighted sum of the corresponding gene expression nodes.


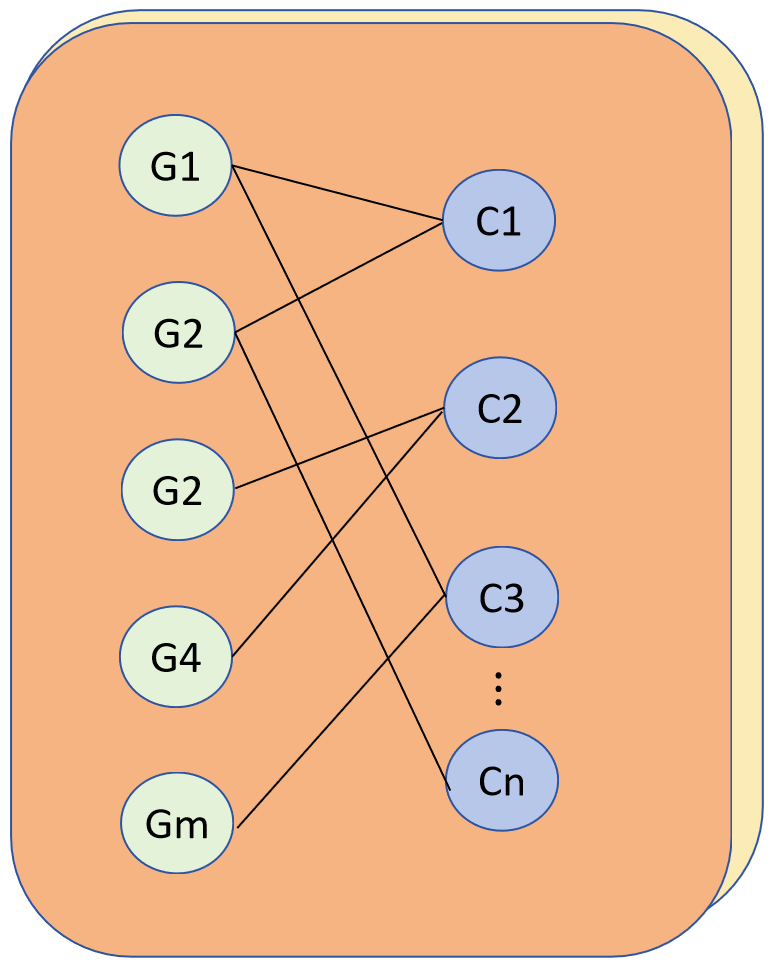


Figure S2. A method for modeling cell-gene interactions in scRNA-seq data using a graph structure. The cell-gene graph models interactions between genes and cells based on the expression matrix, where cells are connected to all expressed genes. Gene nodes are created using the top 2000 principal components of the expression matrix. The encoder maps the relationships between cells and genes into a low-dimensional space, enabling simultaneous processing of both cell and gene nodes.

1. **Evaluation of the Appropriateness of PCA Dimensionality h for Matrix B**

To find the optimal dimensionality that balances information retention and noise reduction, we conduct a series of experiments on the Schyns dataset for various values of h=300,400,…,1200, and plot the curve of ARI as a function of h (Figure S3). The observations are as follows:

(1) When h < 700, as the dimensionality increases, ARI exhibits a clear upward trend, indicating that PCA continues to extract more principal components that contribute to distinguishing cell clusters.

(2) Around h ≈ 700, ARI reaches its peak or plateaus under various configurations. At this point, the reduced features adequately represent gene-cell interaction information while avoiding excessive redundancy or noise.

(3) When h > 700, ARI starts to decline or fluctuate slightly, suggesting that further increasing the dimensionality does not significantly improve clustering quality.

Considering both the stability of the performance curve and computational efficiency, we select h≈700 as the dimensionality for PCA. This value optimally preserves the major variations in gene expression while suppressing redundant noise, providing the best input features for subsequent adjacency matrix construction and graph auto-encoder training.

**
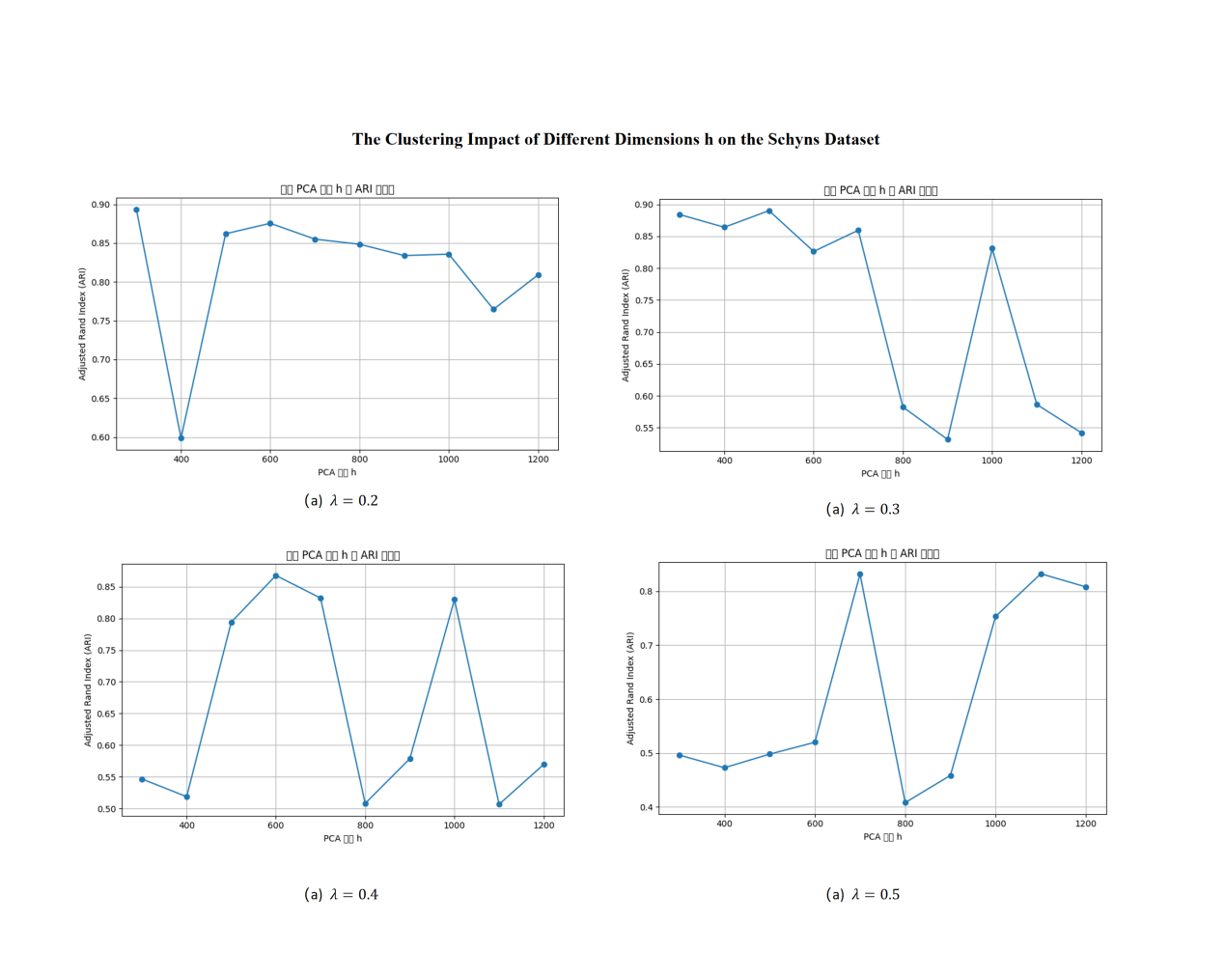
**

Figure S3. This figure illustrates the impact of different dimensionalities h on the clustering performance of the Schyns dataset. Each subplot corresponds to a different λ value (0.2, 0.3, 0.4, 0.5). The x-axis represents the number of principal components retained by PCA (i.e., dimensionality h), and the y-axis shows the value of the Adjusted Rand Index (ARI). The curves in the figure reflect the fluctuations in clustering performance as h changes.

1. **Analysis of the Parameter λ in the Global Cell–Gene Adjacency Matrix**

The complete cell-gene adjacency matrix structure of scGGC is shown in Figure S4. In single-cell data analysis, balancing the relationships between cells and genes is crucial for accurately capturing the complex interactions between them. The weight parameter λ plays a key role in this process by regulating the relative weights of these two relationships in the cell-gene graph structure, ensuring that the model considers both the similarity between cells and the association between genes. Specifically, the value of λ typically ranges from [0, 1], adjusting the weight ratio between the two matrices (C and B) when constructing the complete adjacency matrix A, thereby affecting the model's focus on cell-to-cell and gene-to-gene relationships. Specifically:

1. When λ = 0.5, the relationship between cells and genes is treated equally.
2. When λ approaches 0, the model places more emphasis on the cell-gene relationships.
3. When λ approaches 1, the cell-to-cell relationship becomes the dominant factor.

In the experimental section, we performed a sensitivity analysis on the value of λ. Figure 7 of the paper shows that the clustering performance (e.g., ARI metric) of the model is optimal when λ is between 0.2 and 0.4 across multiple datasets. This suggests that within this range, the model effectively balances the relationships between cells and cell-gene interactions, thus avoiding information loss or overfitting that might occur due to overemphasis on one aspect. For example, in the MHC3K dataset, when λ is 0.3, the ARI reaches 0.81. In the Leary dataset, λ between 0.2 and 0.4 yields the best ARI, and performance begins to decline as λ increases. This further validates the appropriateness of λ within the range of 0.2 to 0.4.

Figure S4. An example of the complete cell-gene adjacency matrix structure. The top-left corner represents the cell-cell adjacency matrix, which captures similarities or correlations between cells. By adjusting the weight coefficients, we can control the influence of cell-cell relationships on the overall structure. The top-right and bottom-left corners are the cell-gene interaction submatrices, representing interactions between cells and genes. By setting $\lambda\in(0,1)$, we ensured a weight balance between the connection strength between cells and genes and the cell-cell relationship. The bottom-right corner represents the gene-gene submatrix. We employ a zero matrix to disregard the independence among genes, thereby facilitating a more accurate capture of the relationships between cells and between cells and genes.

1. **Extension to Large-Scale Datasets**

To address large-scale datasets, we introduced an L2 regularization term based on model parameters into the standard reconstruction loss function. This addition aims to prevent overfitting and further enhance the model’s generalization capability:

${\mathcal{\mathcal{L}}}_{total}={\mathcal{\mathcal{L}}}_{Oringinal} + \xi||W||_{2}^{2}$ (1)

Where, $W$ denotes the model's weight matrix. The L2 regularization term $||W||_{2}^{2}$ constrains the magnitude of the weights to mitigate overfitting caused by excessively large model parameters. The parameter$\xi$serves as a regularization parameter to balance the reconstruction loss and the regularization term.

To ensure fairness in the experimental comparisons, we carefully controlled all variables: L2 regularization was introduced only during the encoder training phase of the scGGC model. All other parameters—including learning rate, layer configuration, and adjacency matrix construction—were kept constant.

| Dataset | Cells | Genes | Cluster | No/Yes（ARI） | No/Yes（FMI） |
| --- | --- | --- | --- | --- | --- |
| 10k_V3 | 11769 | 33538 | 7 | 0.8 / 0.82 | 0.85 / 0.89 |
| Dekoninck | 21589 | 19790 | 19 | 0.59 / 0.62 | 0.61 / 0.68 |
| Pbmc20k | 23837 | 36601 | 10 | 0.71 / 0.77 | 0.79 / 0.82 |
| GSE223414 | 45460 | 25639 | 12 | 0.67 / 0.71 | 0.73 / 0.79 |

Table S1. Clustering performance comparison (in terms of ARI and FMI) before and after introducing L2 regularization across various single-cell datasets.

Under this setting, we evaluated the impact of regularization on clustering performance across multiple real large-scale single-cell datasets. The experimental results (Table S1) demonstrate that incorporating L2 regularization leads to a notable improvement in clustering performance. For instance, in the 10k_V3 dataset, the ARI increased from 0.80 to 0.82; in the Dekoninck dataset, the FMI improved from 0.61 to 0.68; and in the Pbmc20k dataset, the ARI showed a relative increase of 8.5%. Figure S5 presents the clustering visualization results of the Pbmc20k dataset. It can be observed that, after introducing regularization, the cluster structure becomes more distinct and overlapping regions are reduced. These results indicate that regularization effectively enhances the accuracy and robustness of the model in handling high-dimensional sparse data, thus validating the effectiveness of the proposed approach. The sources of the datasets used in this study are detailed in Table S2.


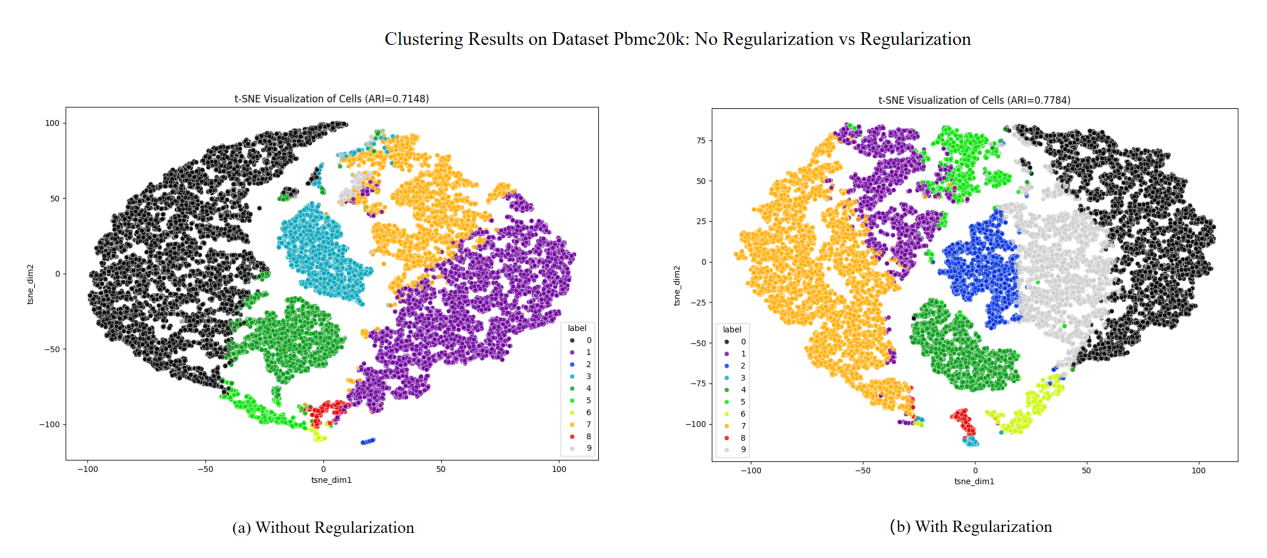


Figure. S5. T-SNE clustering visualizations of the Pbmc20k dataset under (a) without and (b) with L2 regularization. Panel (a) shows the clustering result without regularization, yielding an ARI of 0.7148. Panel (b) presents the result after incorporating L2 regularization, where the ARI increases to 0.7784. Each point represents a cell, and colors indicate different clustering groups.

| Dataset | Source |
| --- | --- |
| 10k_V3 | <https://www.10xgenomics.com/datasets/10-k-pbm-cs-from-a-healthy-donor-v-3-chemistry-3-standard-3-0-0> |
| Dekoninck | <https://www.ebi.ac.uk/gxa/sc/experiments/E-GEOD-146122/results/cell-plots> |
| Pbmc20k | <https://www.10xgenomics.com/datasets/20-k-human-pbm-cs-3-ht-v-3-1-chromium-x-3-1-high-6-1-0> |
| GSE223414 | <https://www.ncbi.nlm.nih.gov/geo/query/acc.cgi?acc=GSE223414> |

Table S2. Sources of the datasets used in this study.

1. **Evaluation Metric Formula**

In the experiments of this study, we employed the Adjusted Rand Index (ARI) to assess the performance of clustering algorithms. The ARI is a widely used metric for evaluating clustering when the true labels are known. It evaluates the clustering effect by measuring the consistency between the predicted clustering labels and the true labels. The formula for calculating ARI is as follows:

$ARI=\frac{\sum_{\mathrm{ij}} (_{2}^{n_{\mathrm{ij}}})-(\sum_{i} (_{2}^{a_{i}}))(\sum_{j} (_{2}^{b_{j}}))}{\frac{1}{2}(\sum_{i} (_{2}^{a_{i}})+\sum_{j} (_{2}^{b_{j}})))-(\sum_{i} (_{2}^{a_{i}}))(\sum_{j} (_{2}^{b_{j}}))}$ (2)

In the contingency table, $n_{\mathrm{ij}}$represents the value in the i-th row and j-th column, that is, the number of sample pairs predicted to be of class i and actually belonging to class is the sum of the i-th row in the contingency table, indicating the total number of samples belonging to class i; $b_{j}$is the sum of the j-th column, representing the total number of samples predicted to be of class j.

Accuracy (ACC), which is used to measure the degree of agreement between clustering results and true labels. The formula for calculating it is as follows:

$ACC=\frac{\sum_{i=1}^{n} 1(y_{i}=\hat{y}_{i})}{n}$ (3)

Where, n is the number of samples, $y_{i}$ is the true label of the i-th sample, $\hat{y}_{i}$ is the predicted label of the i-th sample, and 1 is an indicator function that takes the value of 1 when $y_{i}=\hat{y}_{i}$, and 0 otherwise.

The Fowlkes-Mallows Index (FMI) is a comprehensive measure of the precision and recall of clustering results, analogous to the F1 score. The formula for its calculation is as follows:

$FMI=\frac{1}{n}\sum_{i,j} \sqrt{\frac{TP_{\mathrm{ij}}}{(TP_{\mathrm{ij}}+FP_{\mathrm{ij}})(TP_{\mathrm{ij}}+FN_{\mathrm{ij}})}}$ (4)

Where, $TP_{\mathrm{ij}}$ represents the number of samples that are truly labeled as class i and predicted as class j, $FP_{\mathrm{ij}}$ represents the number of samples that are not truly labeled as class i but are predicted as class j, and $FN_{\mathrm{ij}}$ represents the number of samples that are truly labeled as class i but are predicted as class j.

Normalized Mutual Information (NMI) is a measure of the amount of shared information between clustering results and true labels. Its calculation formula is as follows:

$NMI(U,V)=\frac{I(U,V)}{\sqrt{H(U)\cdot H(V)}}$ (5)

Where, $I(U,V)$ is the mutual information between the clustering result U and the true labels V, measuring the amount of shared information between them, and is formulated as:

$I(U,V)=\sum_{i=1}^{k} \sum_{j=1}^{k^{'}} p(u_{i},v_{j})log\frac{p(u_{i},v_{j})}{p(u_{i})p(v_{j})}$ (6)

Where, $u_{i}$and$v_{j}$represent the cluster labels and true labels, respectively. $p(u_{i},v_{j})$ is the probability that label $u_{i}$ from clustering result U and label$v_{j}$from true label V appear simultaneously. $p(u_{i})$and $p(v_{j})$ are the marginal probabilities of the cluster labels and true labels, respectively.

The evaluation results of the data set are shown in Table S3

| Dataset | Cells | Cluster | ARI | ACC | NMI | FMI |
| --- | --- | --- | --- | --- | --- | --- |
| **pbmc4k** | 4340 | 7 | 0.81 | 0.87 | 0.84 | 0.86 |
| **MKTA1K** | 1385 | 9 | 0.71 | 0.74 | 0.75 | 0.72 |
| **Kasper** | 4351 | 9 | 0.75 | 0.79 | 0.81 | 0.81 |
| **Shiokawa** | 4449 | 10 | 0.63 | 0.75 | 0.65 | 0.68 |
| **Sun** | 6360 | 7 | 0.83 | 0.87 | 0.82 | 0.88 |
| **Leary** | 1489 | 12 | 0.66 | 0.76 | 0.76 | 0.75 |
| **Schyns** | 6666 | 8 | 0.92 | 0.91 | 0.85 | 0.94 |
| **MHC3K** | 3282 | 8 | 0.89 | 0.88 | 0.86 | 0.91 |
| **Efremona** | 2130 | 13 | 0.74 | 0.79 | 0.81 | 0.76 |

Table S3. Comparison of clustering metrics.

1. **High-Confidence Sample Selection**

Each cell's features are represented as $Z_{i}$ (where i denotes the i-th cell). We perform K-means clustering based on the embedding vector Z. For all cells $Z_{1}$,$Z_{2}$,…$Z_{m}$in a cluster $C_{k}$(where mm is the number of samples in the cluster), we compute the centroid $\mu_{k}$ of the cluster. The formula for the centroid is as follows:

$\mu_{k}=\frac{1}{m}\sum_{i=1}^{m} Z_{i}$ (7)

Next, we calculate the Euclidean distance between each cell $x_{i}$ and the centroid $\mu_{k}$ of its assigned cluster. This distance metric reflects the similarity or compactness of each cell relative to the cluster center. The Euclidean distance is computed using the following formula:

$d(z_{i},\mu_{k})=\sqrt{\sum_{j=1}^{d} {(Z}_{i,j}-\mu_{k,j})^{2}}$ (8)

Where, d represents the dimensionality of the feature space, and $Z_{i,j}$ and $\mu_{k,j}$ denote the values of the i-th cell and the centroid in the j-th feature dimension, respectively.

To select high-confidence samples, we use the distance of each cell to its centroid. To ensure the selection of cells that best represent the cluster, we choose the top p% of cells with the smallest distances to the centroid within each cluster. The indices of these high-confidence samples are given by:

$x_{\mathrm{real}}=\left\{ x_{i}|i \epsilon argsort(d(x_{i},\mu_{k}))[:top_{p}] \right\}$ (9)

Where, $argsort(d(x_{i},\mu_{k}))$returns the indices of cells sorted by their distance to the centroid in ascending order, and $x_{\mathrm{real}}$ represents the selected high-confidence samples, typically the top p% of cells.

During the experiment, we also attempted a random selection method for each cluster, but it failed to achieve the desired results in terms of clustering stability and biological relevance. Figure S6 displays a comparison of the random selection method with the centroid distance-based high-confidence sample selection method across multiple evaluation metrics. In contrast, the centroid distance-based high-confidence sample selection method demonstrated greater stability, contributing to the generation of more reliable clustering results and further enhancing the clustering performance of the model.


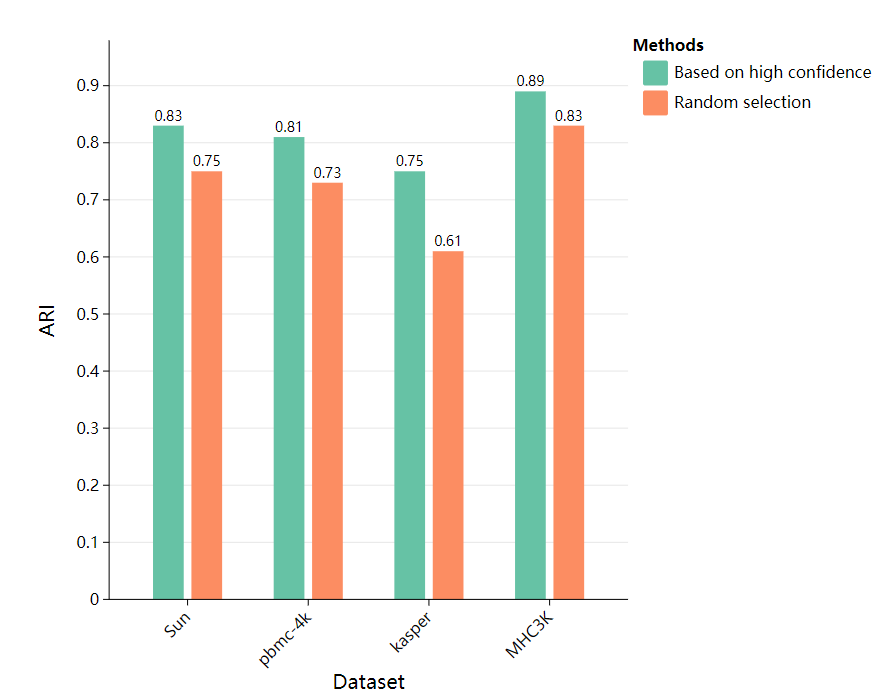


Figure S6. The figure presents a comparison of the ARI evaluation metric between the random selection method and the centroid distance-based high-confidence sample selection method across multiple datasets (Sun, MHC3K, pbmc-4k, Kasper)

1. **Dataset Clustering Visualization**


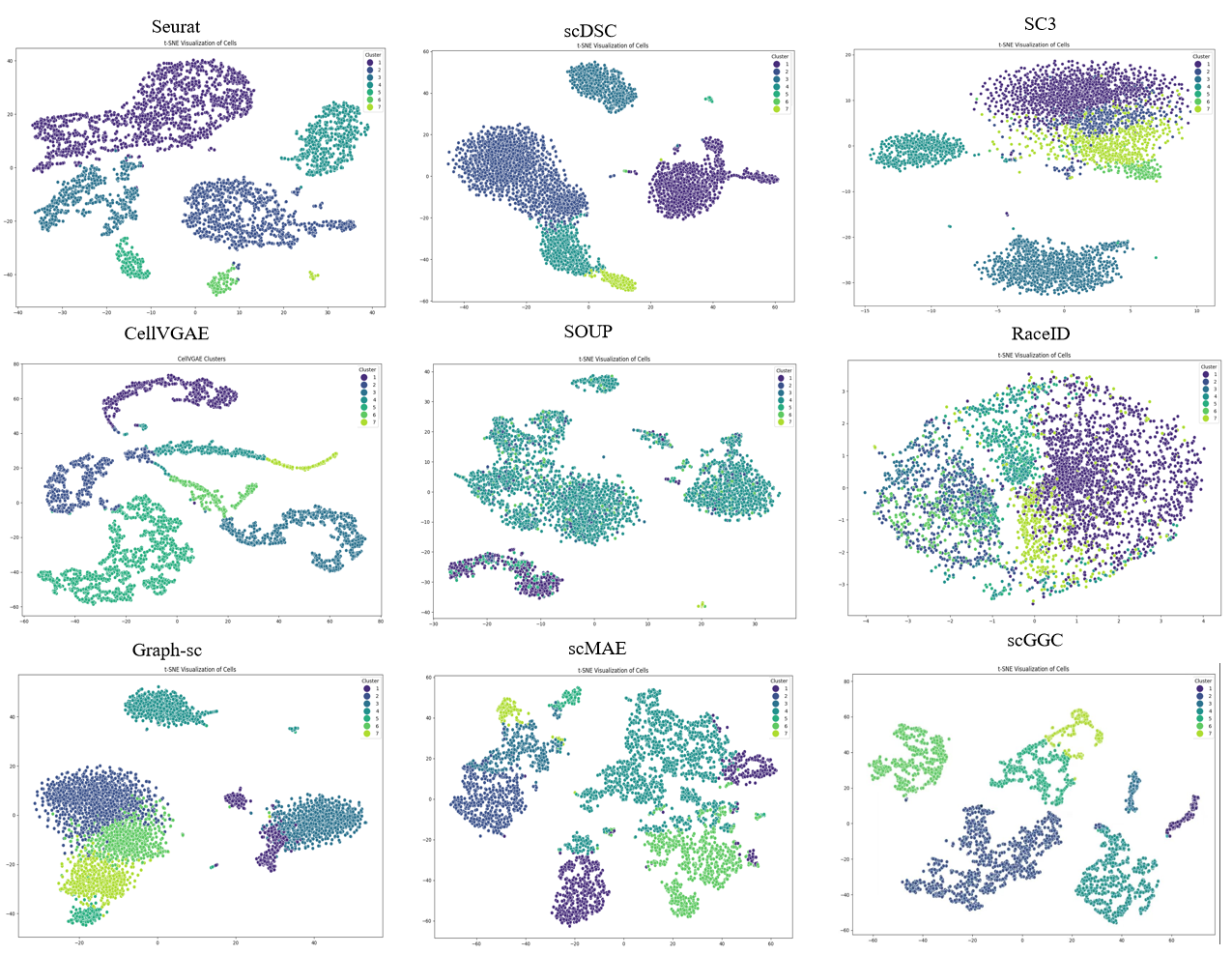


Figure S7. T-SNE plots of cell distribution in the pbmc4k dataset under nine clustering methods.


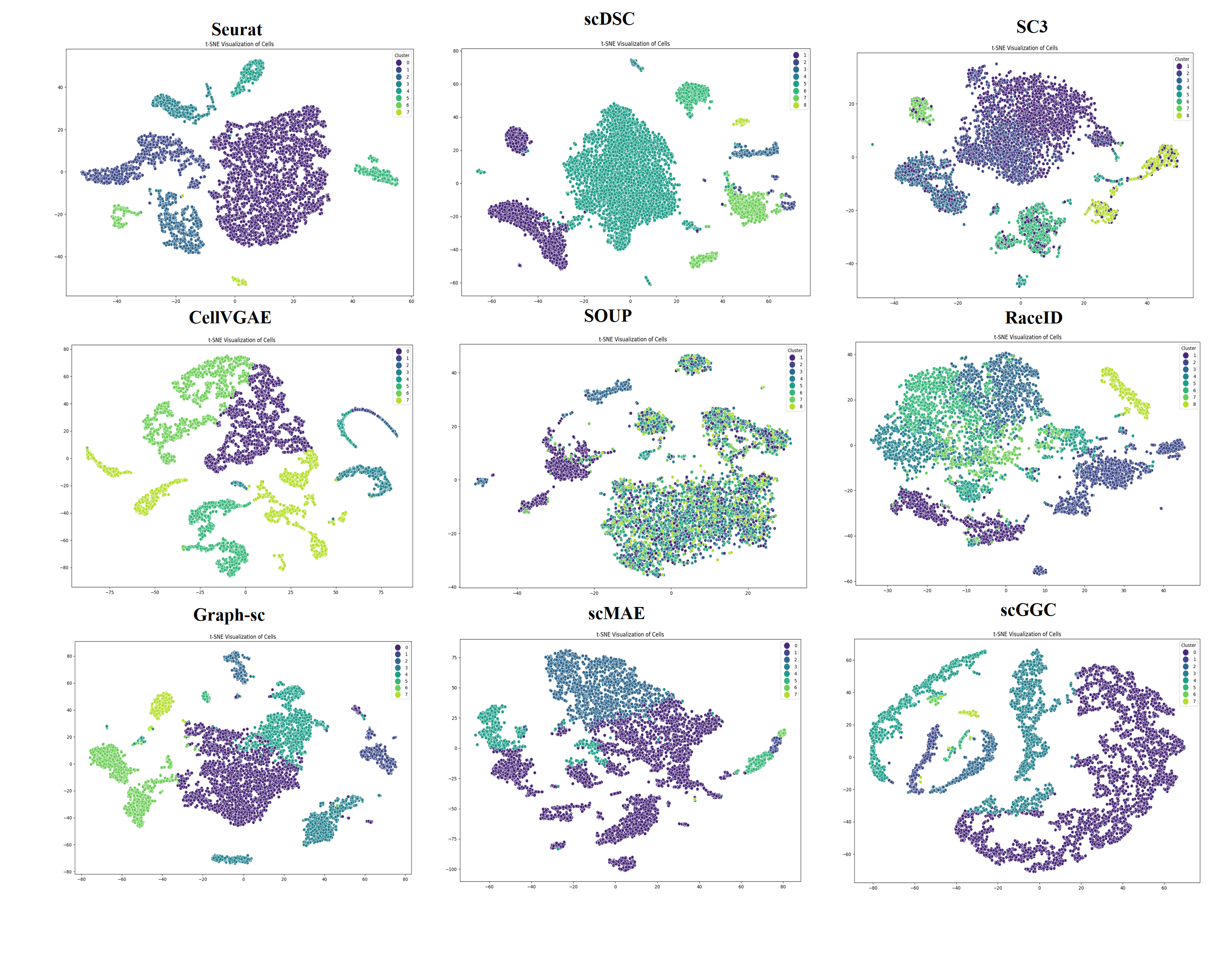


Figure S8. T-SNE plots of cell distribution in the Schyns dataset under nine clustering methods.


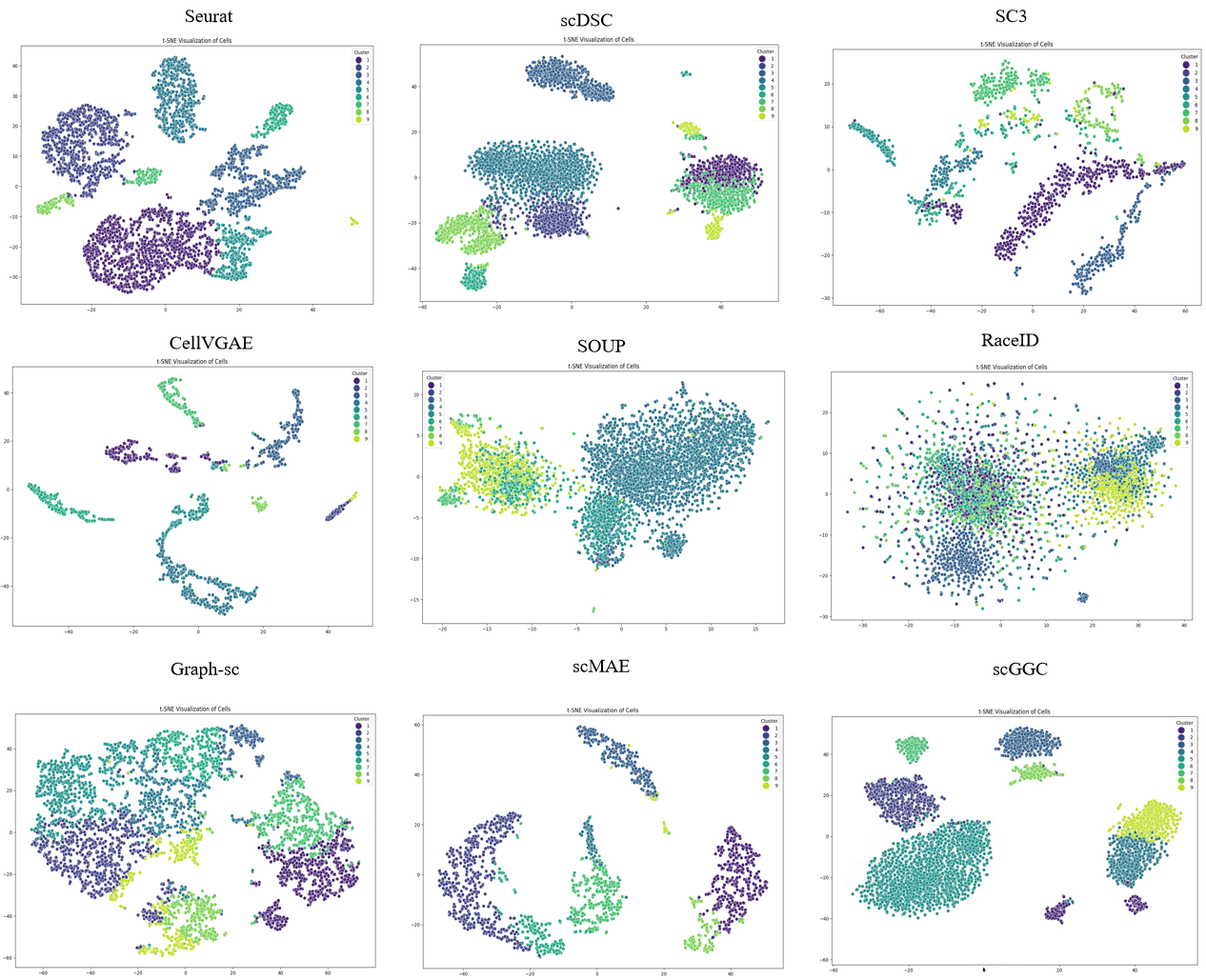


Figure S9. T-SNE plots of cell distribution in the Kasper dataset under nine clustering methods.


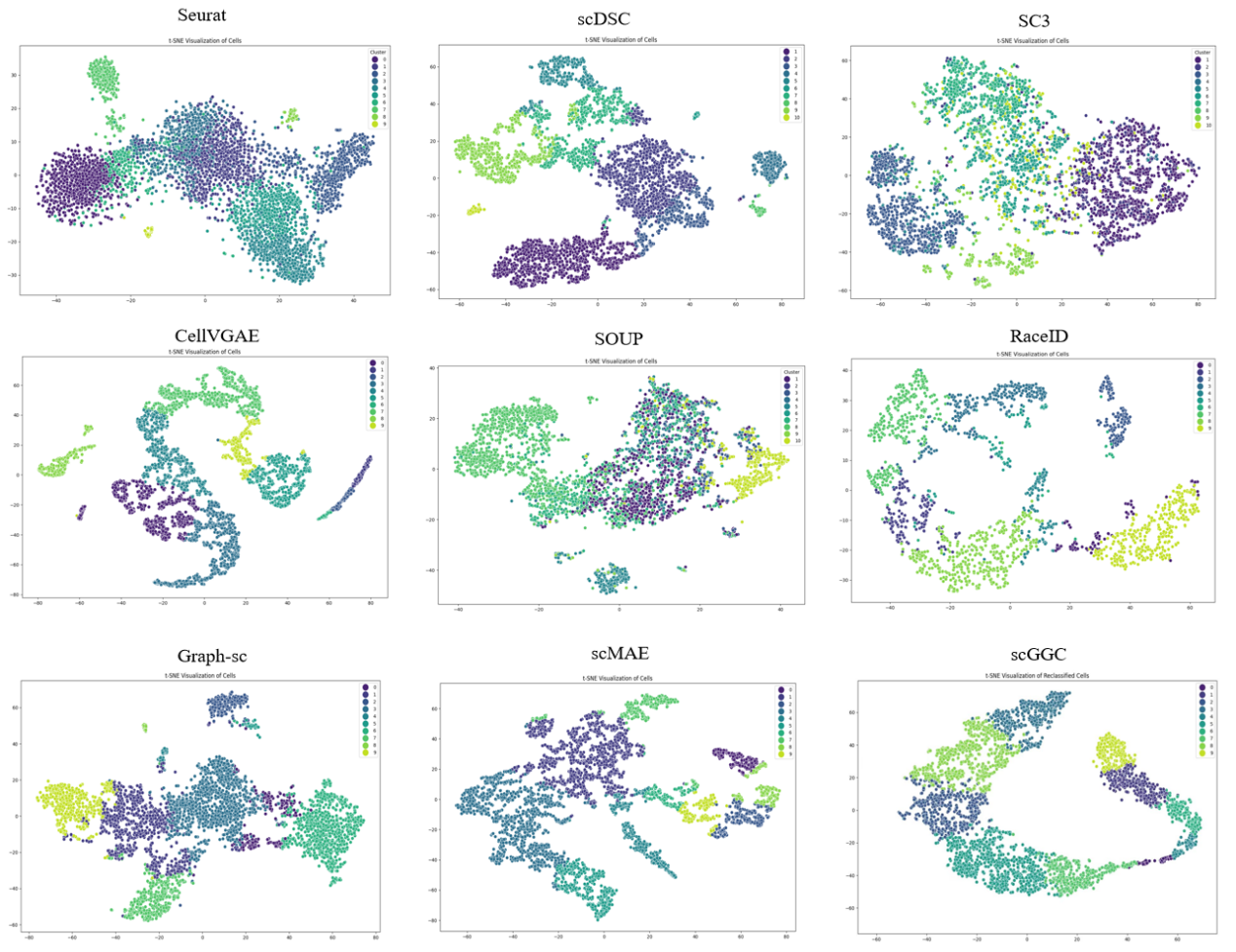


Figure S10. T-SNE plots of cell distribution in the Shiokawa dataset under nine clustering methods.


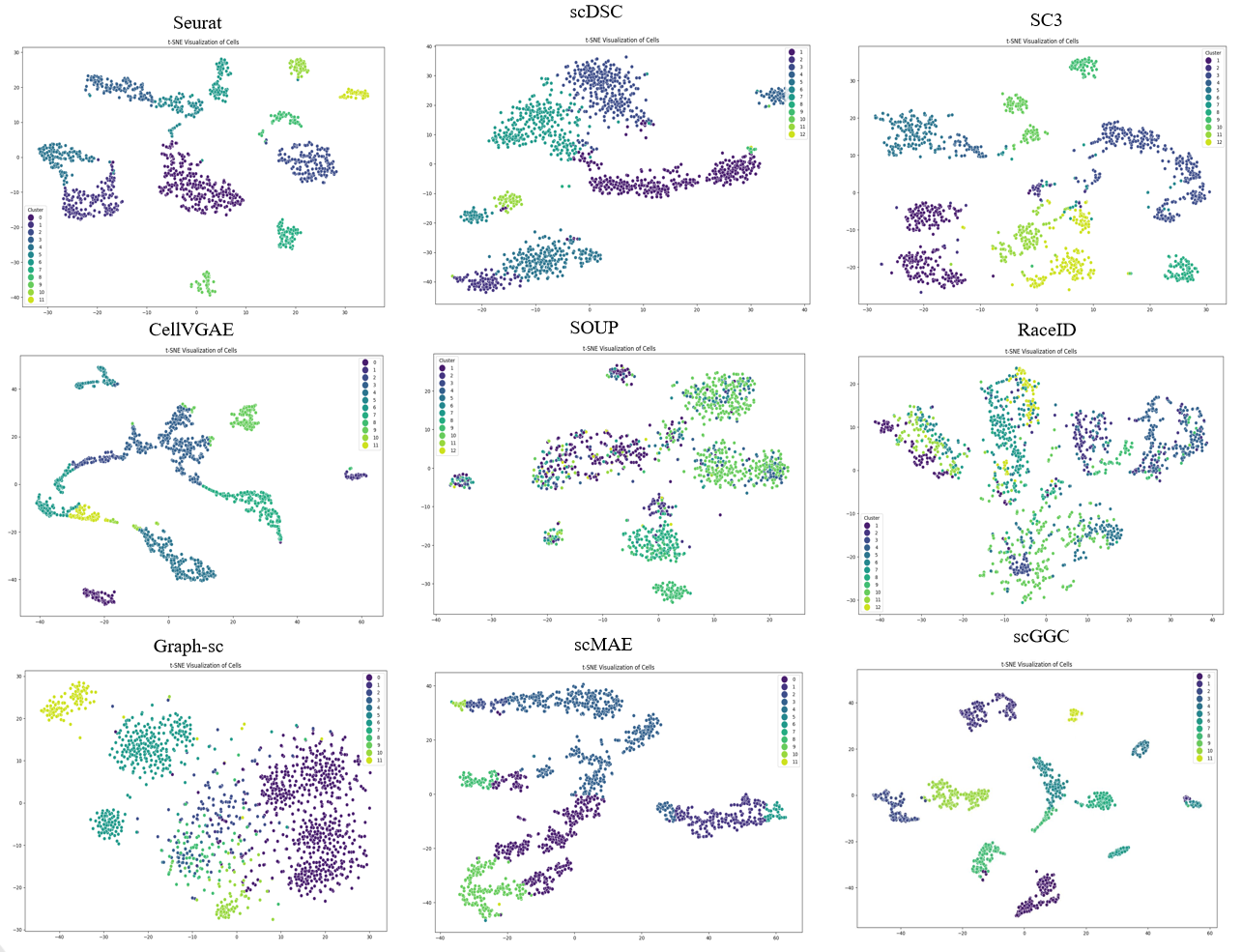


Figure S11. T-SNE plots of cell distribution in the Leary dataset under nine clustering methods.


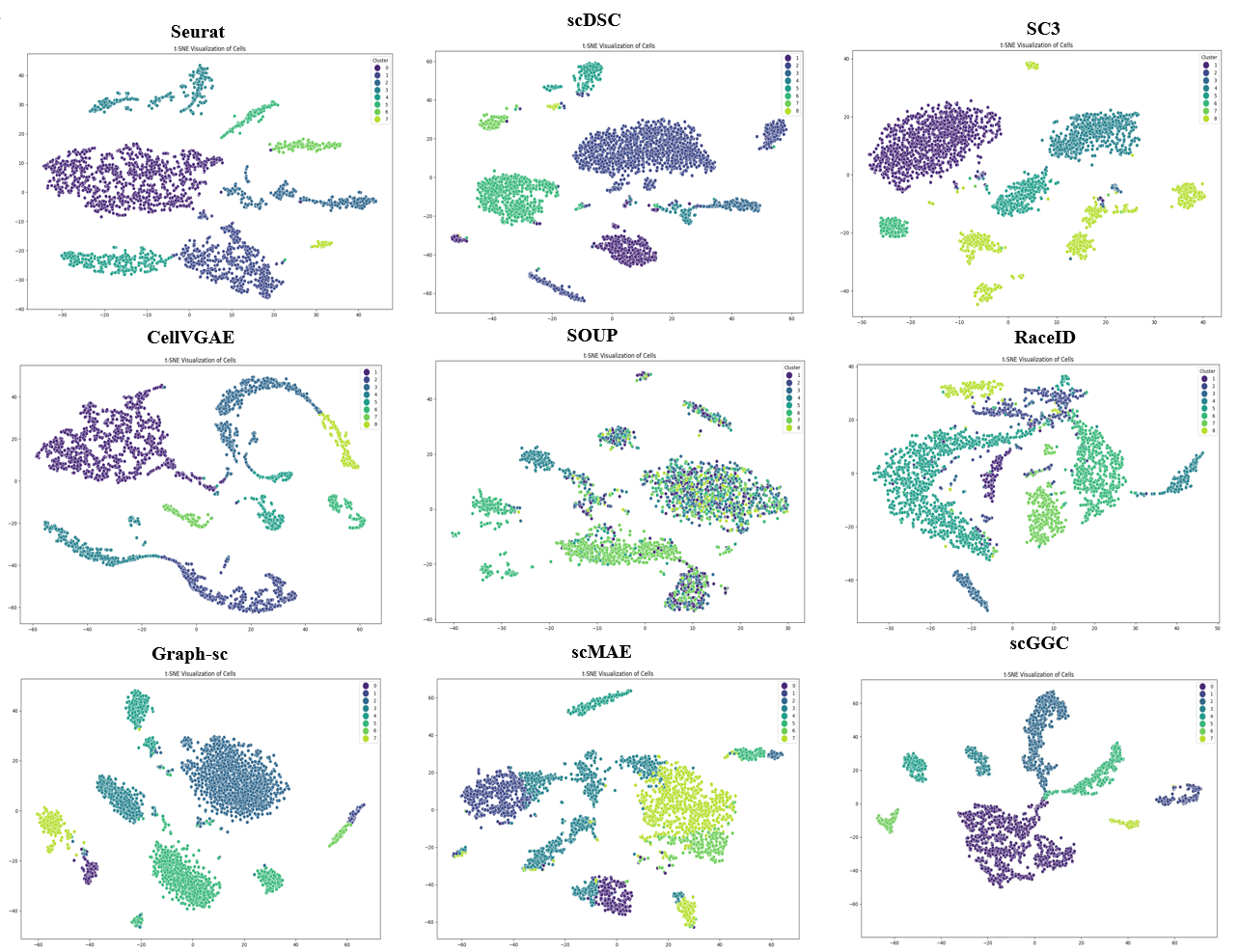


Figure S12. T-SNE plots of cell distribution in the MHC3K dataset under nine clustering methods.


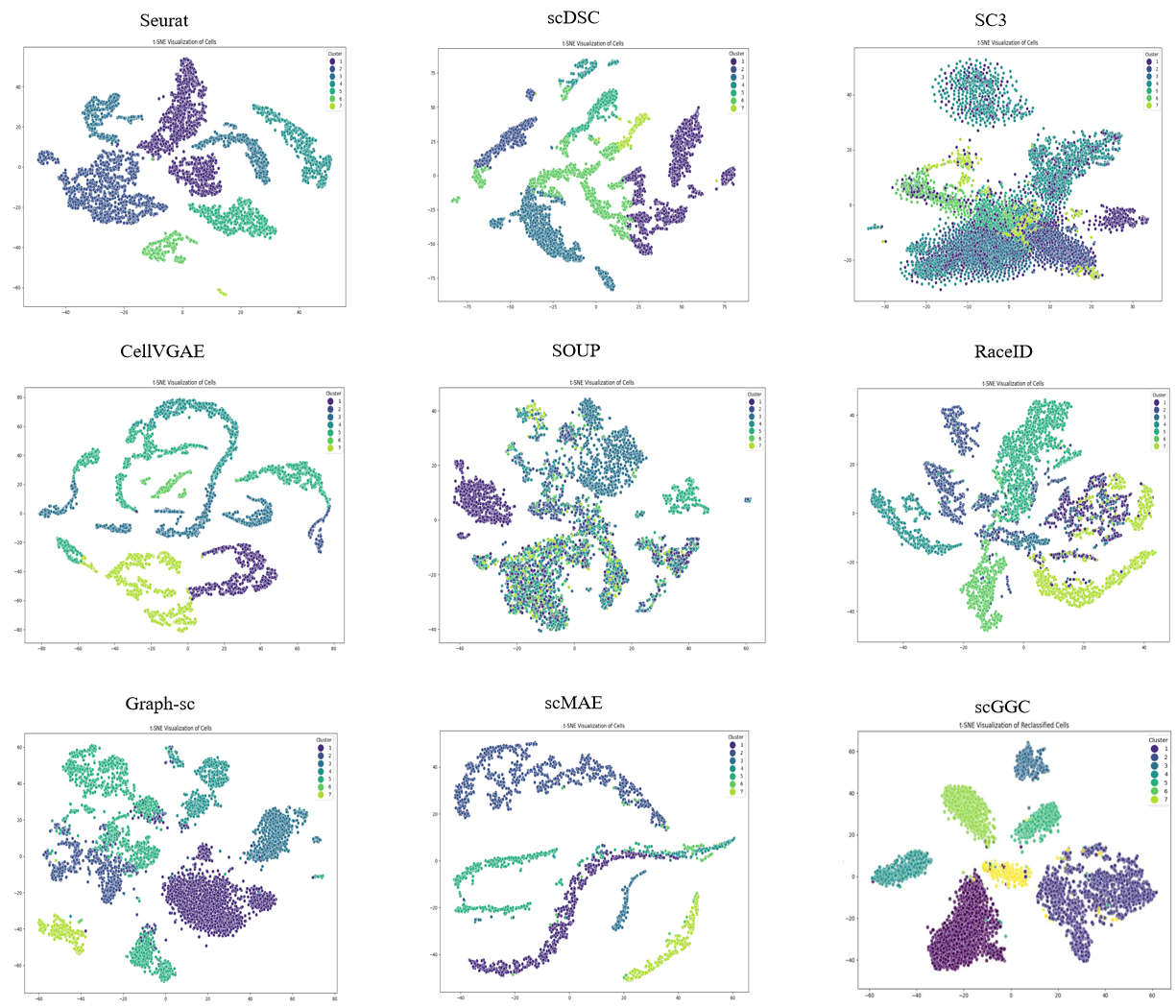


Figure S13. T-SNE plots of cell distribution in the Sun dataset under nine clustering methods.


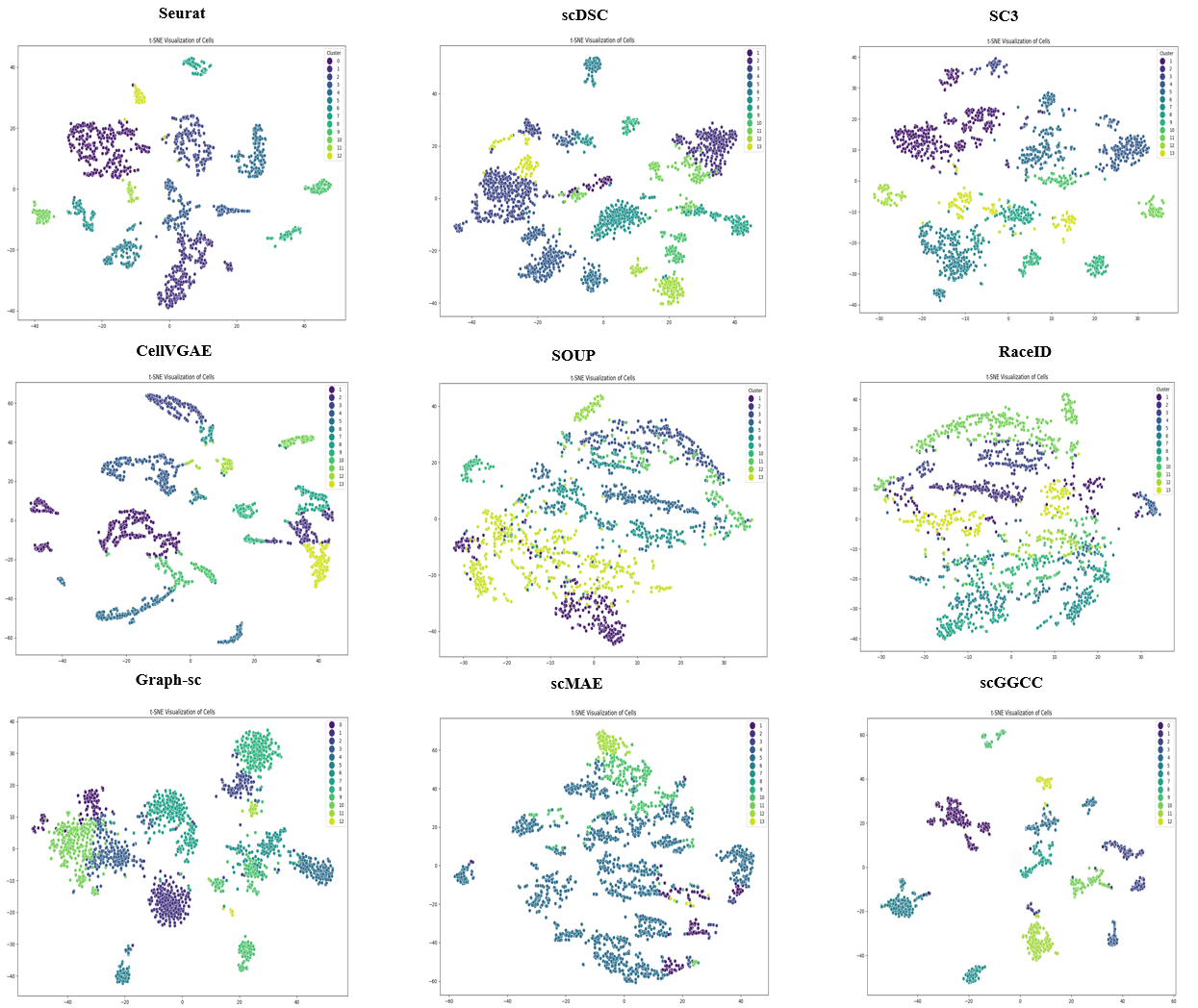


Figure S14. T-SNE plots of cell distribution in the Efremova dataset under nine clustering methods.

1. **Visualization of Marker Gene Heatmaps**


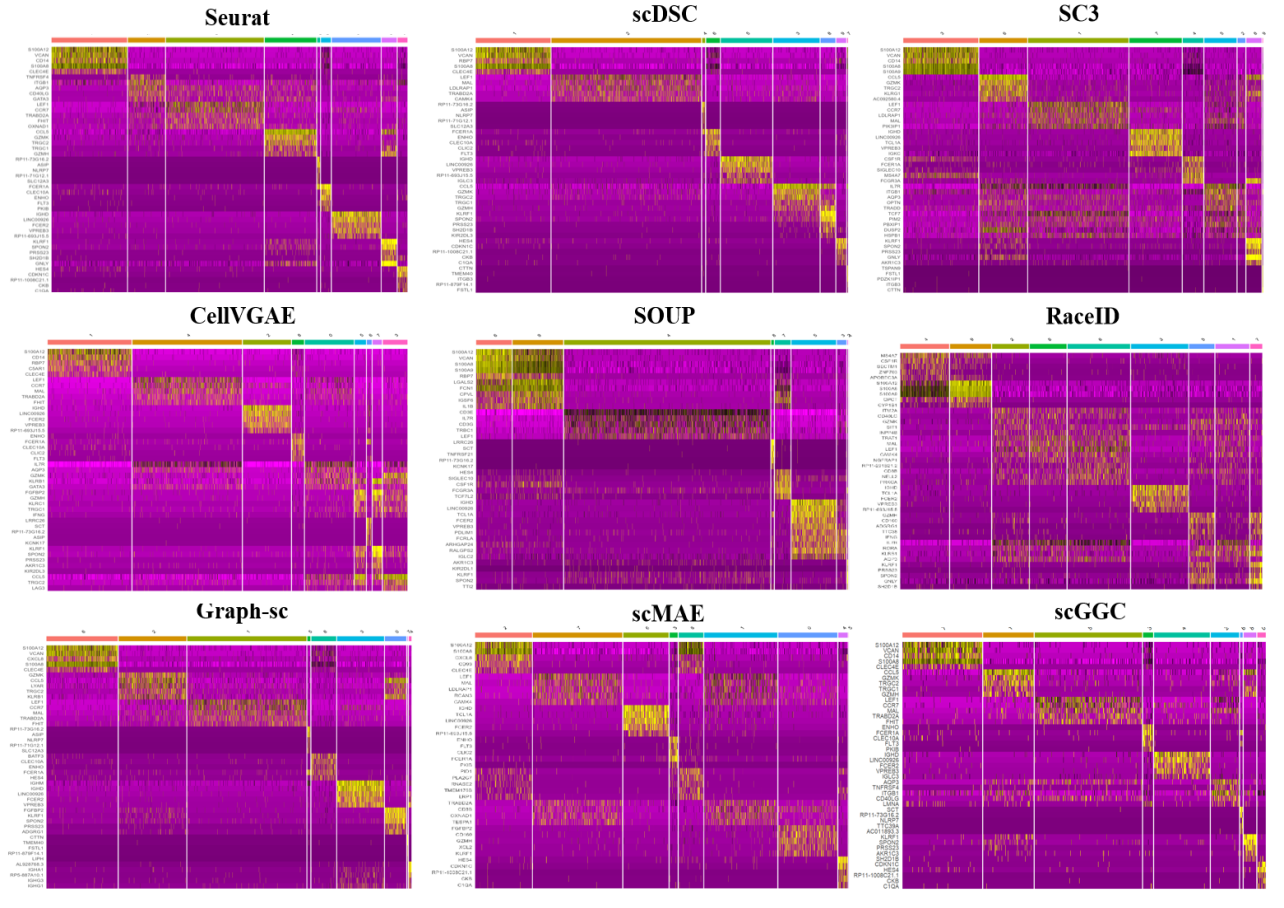


Figure S15. Heatmap of marker gene distributions for each subgroup in the pbmc4k dataset obtained using scGGC and eight other clustering methods.


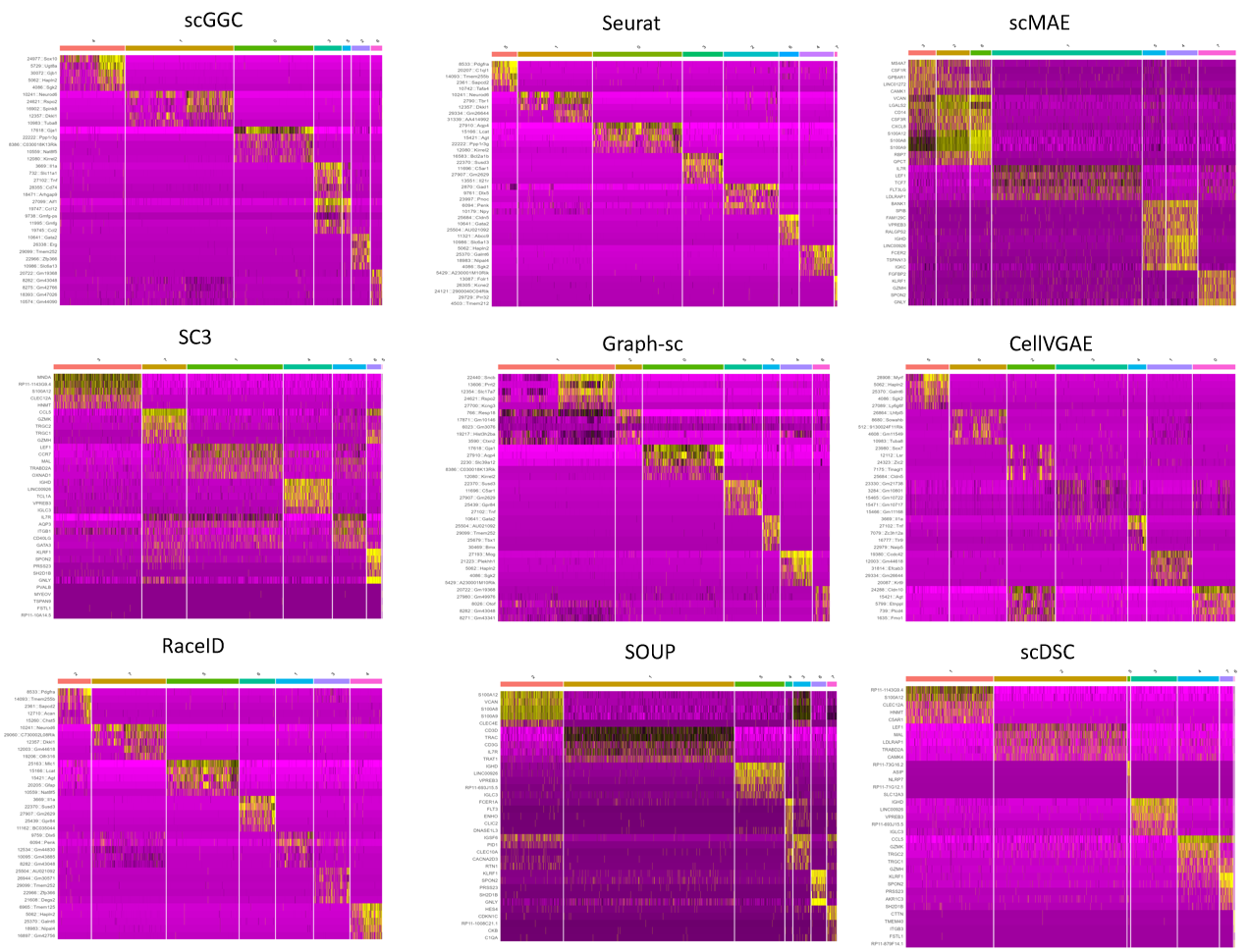


Figure S16. Heatmap of marker gene distributions for each subgroup in the Sun dataset obtained using scGGC and eight other clustering methods.

1. **Biological Validation of Clustering Analysis**

Using the Schyns dataset as an example, we performed marker gene analysis for each cluster using the FindAllMarkers function. Figure S17 intuitively illustrates the heatmap of the top five marker genes identified in each cluster. As shown in the figure, these marker genes (e.g., C1qa, Sirpd, Clnd5, Upk3b) exhibit high expression levels in specific clusters and are closely associated with the biological functions of particular cell types.


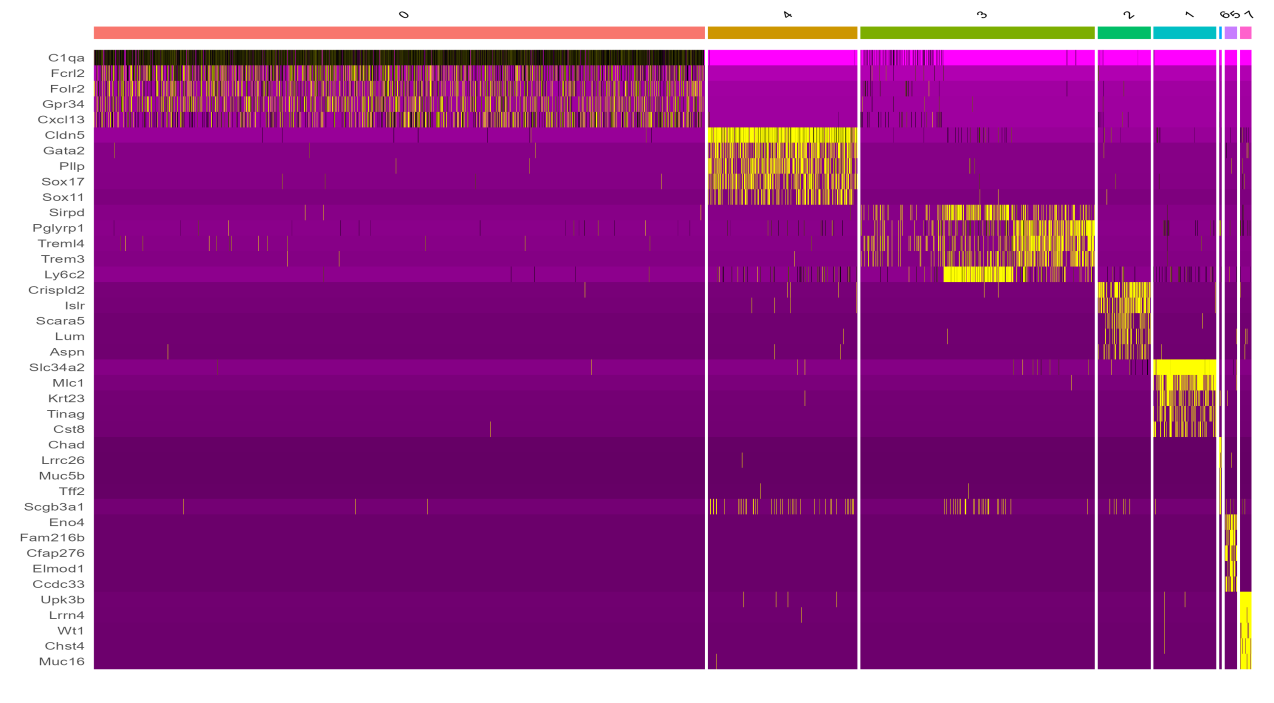


Figure S17. Expression patterns of selected genes across cell clusters in the Schyns dataset. Rows represent genes, and columns represent cell clusters. The color intensity reflects the expression level of each gene, with darker colors indicating higher expression and lighter colors indicating lower expression.

Subsequently, we performed cell type annotation based on the ImmGen reference database. As shown in Figure S18(a), the t-SNE visualization illustrates the distribution of clusters, while Figure S18(b) displays the annotated cell types. A comparison between the two reveals that the purple cluster (Cluster 0) predominantly corresponds to macrophages, and the red cluster (Cluster 7) overlaps significantly with the distribution of urothelial cells, further validating the biological relevance of the clustering results.

**
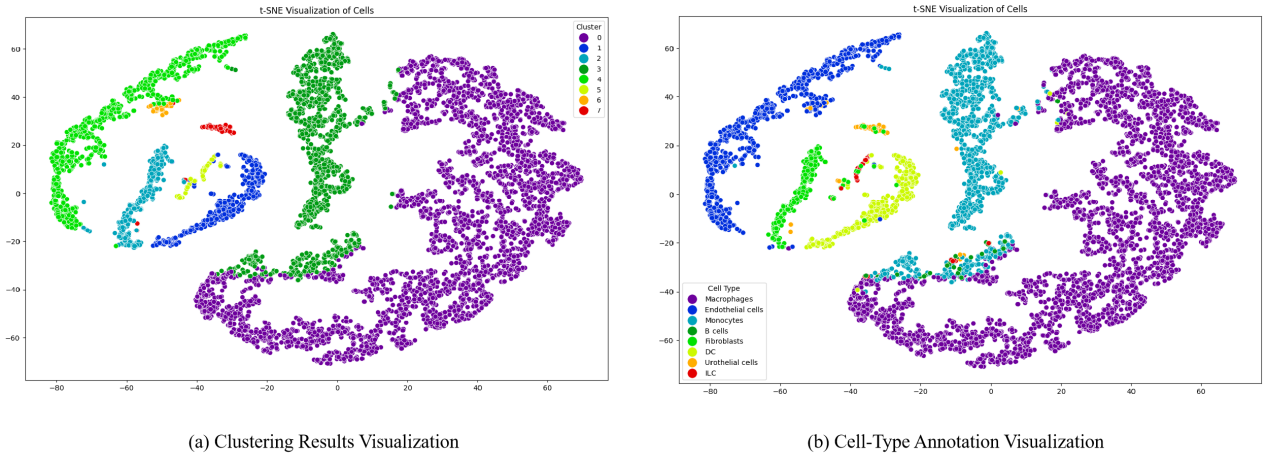
**

Figure S18. (a) t-SNE clustering visualization of the dataset, where points in different colors represent different clusters (Cluster 0 to Cluster 7). (b) t-SNE visualization after cell type annotation, where points in different colors represent different cell types (including endothelial cells, macrophages, etc.). A comparison between the two figures allows observation of the correspondence between clustering results and cell types, validating the biological relevance of the clustering analysis.

To clearly present the high expression levels of marker genes in cells, we selected a subset of marker genes to create Figure S19. By comparing the results with cell type annotations (Figure S18(b)), we found that the regions of high expression of these marker genes were highly consistent with the distribution of specific cell types. Research has shown that C1qa is a stable marker for tissue macrophages and plays a key role in antimicrobial pathways[[1](#_ENREF_1)]; Sirpd is significantly expressed in tumor-associated myeloid cells, especially in monocytes, where it plays an important role in immune checkpoint regulation[[2](#_ENREF_2)]; Cldn5 is highly expressed in endothelial cells of the lungs and brain and is an essential tight junction protein for maintaining vascular barrier function[[3](#_ENREF_3)]; Upk3b is highly expressed in urothelial cells of the normal human bladder and is a key marker gene for identifying urothelial cells[[4](#_ENREF_4)].

**
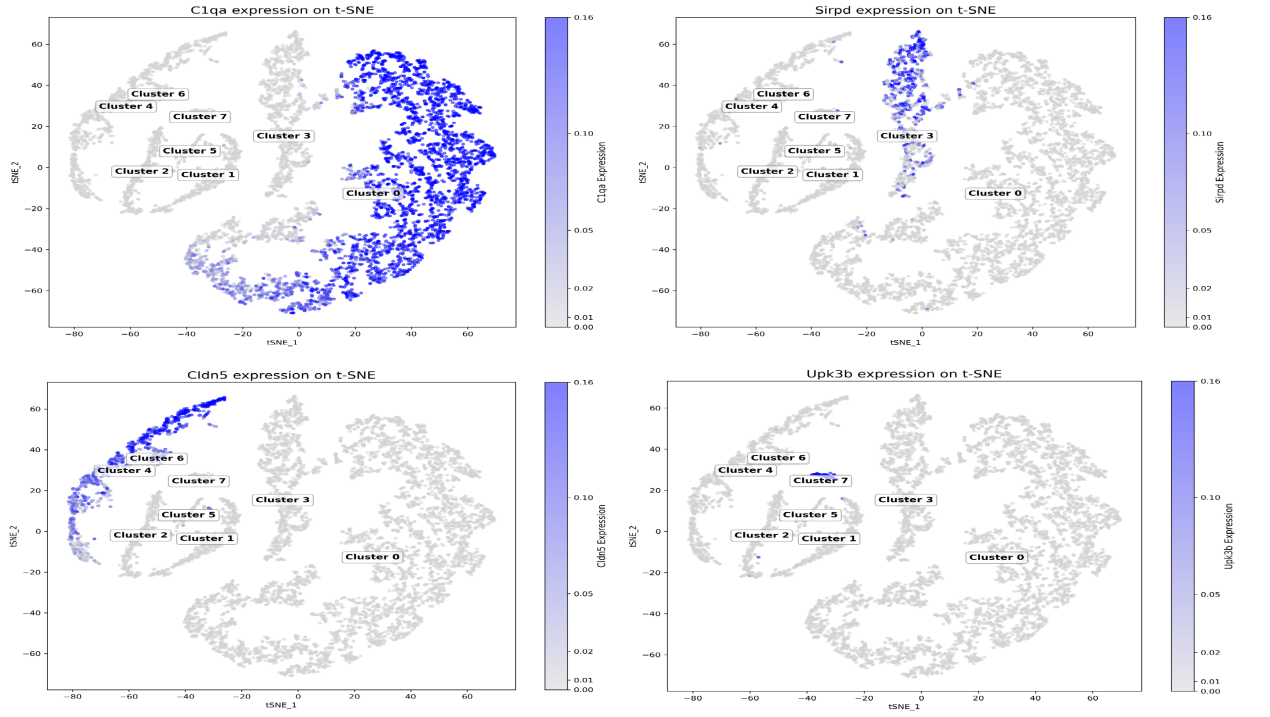
**

Figure S19. Shows the t-SNE visualization of the expression of marker genes (C1qa, Sirpd, Cldn5, Upk3) in cell clusters, where the color intensity represents the gene expression levels. By comparing this figure with the cell type annotation visualization, the results demonstrate a high consistency between the selected marker genes and cell types. This finding strongly suggests that the visualization analysis of these key marker genes can further validate the significant biological relevance between the clustering results and the biological functions of the cell types.

Finally, in the section "Identification of Auxiliary Marker Genes," we calculated the overlap rate between the auxiliary marker genes and the true marker genes by comparing them. This allowed us to quantify the reliability of the clustering results. The multi-level validation presented above clearly demonstrates that the clustering strategy in this study can accurately reflect the cell type-specific transcriptomic features, significantly enhancing the biological credibility of the clustering analysis.


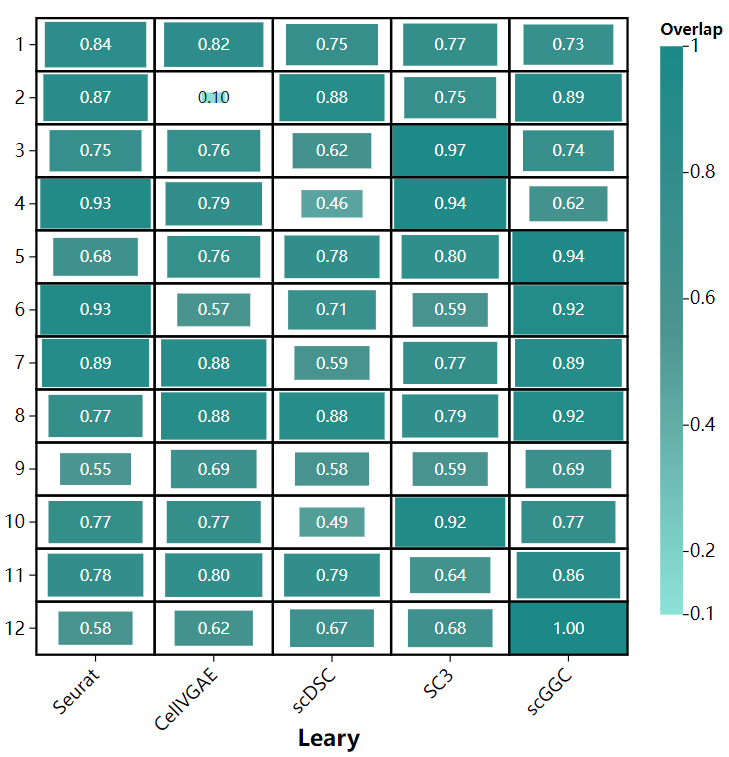


Figure S20. A comparative analysis of marker gene overlap rates was conducted for the Leary datasets, presenting the overlap rates for each cluster. The horizontal axis represents the five methods, and the vertical axis represents the overlap rate corresponding to each cluster.


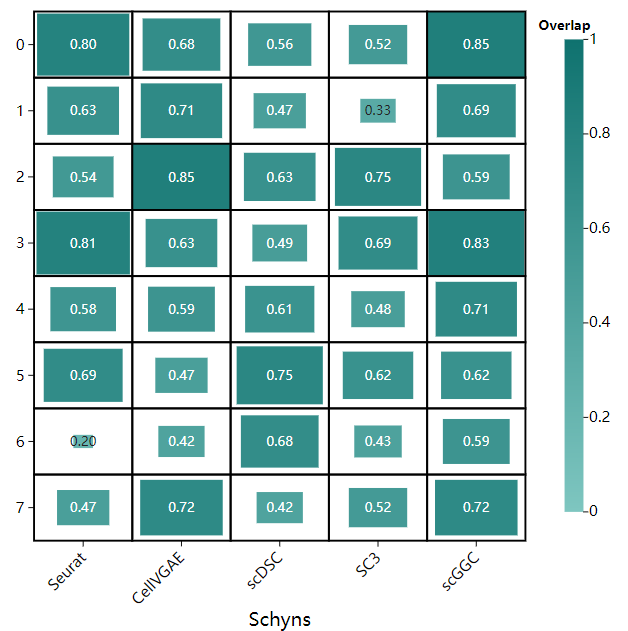


Figure S21. A comparative analysis of marker gene overlap rates was conducted for the Schyns datasets, presenting the overlap rates for each cluster. The horizontal axis represents the five methods, and the vertical axis represents the overlap rate corresponding to each cluster.


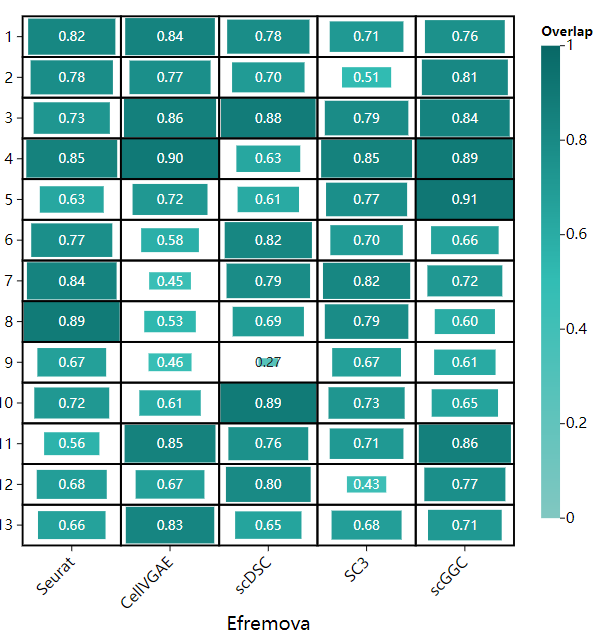


Figure S22. A comparative analysis of marker gene overlap rates was conducted for the Efremova datasets, presenting the overlap rates for each cluster. The horizontal axis represents the five methods, and the vertical axis represents the overlap rate corresponding to each cluster.

1. **Code Repository Instructions**

| Type | Description |
| --- | --- |
| Clone | git clone <https://github.com/Zhi1002/scGGC.git> |
| README.md | Description of the Program Framework and Workflow |
| processing.py | The data preprocessing module is responsible for preprocessing the gene expression data, including data filtering, normalization, and dimensionality reduction operations. |
| models.py | The model definition module implements the structure of the Graph Convolutional Autoencoder (GCNAE) and Generative Adversarial Network (GAN), including components such as the encoder, decoder, generator, and discriminator. |
| scGGC.py | The main script integrates core processes such as model initialization, training, and clustering analysis, serving as the entry point for running the single-cell clustering method. |
| select.py | Select high-confidence samples. |
| training.py | The model training module defines the training process for the graph convolutional autoencoder and generative adversarial network, including the computation of loss functions, parameter updates, and the training loop logic. |
| Environment | Python：3.10.16 |
| Packages | PyTorch：1.13.1+cu116； NumPy：1.26.4  pandas：2.2.3； scikit-learn：1.5.2 |
| Example dataset | Schyns |

Table S4. Overview of the scGGC Code Repository.

1. **Multi-Omics Research: A Glimpse into Future Work**

**Model Extension for Multi-Omics Integration.**

1. Construction of a Unified Adjacency Matrix

To accommodate multi-omics data, we construct individual cell-cell adjacency matrices from single-cell transcriptomic (scRNA-seq), ATAC-seq, and roteomic data, denoted as A1, A2, A3, respectively. These matrices are then integrated into a unified adjacency matrix A through a weighted combination. The construction procedure is as follows:

- The transcriptomics-based cell-cell adjacency matrix A1, as well as the cell-gene bipartite graph B, are constructed following the same procedure as in the original scGGC framework.
- Adjacency Matrix from ATAC-seq Data

To construct the adjacency matrix based on ATAC-seq data, we first utilize a gene annotation file (GTF) to associate open chromatin peaks with the upstream and downstream regions of transcription start sites (TSS) for each gene. Through overlap analysis, we count the number of accessible regions associated with each gene, resulting in a gene activity matrix, where N is the number of cells and M is the number of genes.

$G_{i,j=}\sum_{k \in peaks overlapping with gene j} ATAC_{i}(k)$ (10)

Where, $\mathrm{ATA}C_{i}$(k) denotes the chromatin accessibility of cell i at peak region k, typically quantified by the number of sequencing fragments.

Subsequently, we perform a logarithmic transformation $log(x+1)$on the gene activity matrix and select highly variable genes (HVG) based on the variance - mean relationship. Then, standardization (Z-score normalization) was carry out to eliminate technical biases. Next, we apply principal component analysis (PCA) to reduce the dimensionality of the gene activity matrix and obtain characteristic expressions $X_{2}$. Finally, based on the reduced-dimensionality features, the k - nearest neighbor algorithm (KNN) was employ to construct the adjacency relationships between cells, resulting in the ATAC adjacency matrix A2.

- Construction of Adjacency Matrix Based on Proteomic (ADT) Data

For the proteomic data, we first apply Centered Log-Ratio (CLR) normalization to eliminate the proportional effects of protein expression across different proteins. CLR is a commonly used normalization method for data with compositional properties, effectively reducing the impact of expression differences between proteins.

$\mathrm{CL}R_{(x_{\mathrm{ij}})}=log_{2}(x_{\mathrm{ij}}+1)-log_{2}(g_{j})$ (11)

Where $x_{\mathrm{ij}}$ represents the expression level of protein i in cell j, and $g_{j}$ is the geometric mean of cell j:

$g_{j}=(\prod_{i} (x_{\mathrm{ij}}+1))^{1/p}$ (12)

Where, $p$represents the number of protein features. After CLR transformation, the proportional effects between different proteins are eliminated, enhancing the comparability of the data. Next, we perform PCA on the CLR-transformed data to obtain a lower-dimensional representation.

Finally, we use the KNN algorithm to construct the cell-to-cell adjacency matrix.

- The three adjacency matrices are weighted and fused to obtain the overall cell-to-cell adjacency matrix $C$ :

$C=\varphi_{1}A_{1}+\varphi_{2}A_{2}+\varphi_{3}A_{3}$ (13)

To ensure that the weight thresholds remain within a reasonable range, we set$\varphi_{1}$+$\varphi_{2}$+$\varphi_{3}$=1.

- Finally, based on the cell–cell adjacency matrix $C$ and the cell–gene adjacency matrix B, we construct the overall cell–gene adjacency matrix $A$, such that:

$\begin{matrix} & \text{N M} \\ \begin{matrix} N \\ M \end{matrix} & \left[ \begin{matrix} \lambda\cdot C & (1-\lambda)\cdot B^{T} \\ (1-\lambda)\cdot B & 0 \end{matrix} \right] \end{matrix}=A$ (14)

1. Feature Information Integration

To fully integrate multi-modal feature expression information, we concatenate the feature representations X1, X2, X3 along the feature dimension to form a unified overall feature representation X:

$X=[X_{1},X_{2},X_{3}]$ (15)

1. Graph Auto-encoder Training and Centroid Calculation

We adopt the models illustrated in Fig. 1(b) and Fig. 1(c) of scGGC. First, the complete adjacency matrix A and the feature representation matrix X are fed into a graph auto-encoder for training to obtain the embedding vectors Z. The embedding vectors Z are then clustered (e.g., using Louvain or k-means), yielding a cluster label for each cell. Next, for the set of cell indices within cluster k, we compute the cluster centroid:

$\mu_{k}=\frac{1}{{|S}_{k}|}\sum_{i\epsilon S_{k}} z_{i}$ (16)

At the current stage, we have achieved a deep integration of multi-omics information at both the structural level (adjacency matrix) and the feature level (expression matrix), resulting in a unified fused embedding representation. In future work, we plan to explore a confidence evaluation mechanism based on this integrated embedding, aiming to further investigate its potential for assessing sample reliability in the context of multi-dimensional feature integration.

**Improvements on Confidence Scoring:** It is important to emphasize that the embedding vectors used here are not derived from a single modality, but are the result of a coordinated integration of multi-omics data, endowing the resulting confidence scores with a comprehensive representational capacity rooted in multi-source information fusion.

1. Centroid Distance Score:

$S_{i}^{dist}=\frac{1}{1+exp(\gamma*dist(z_{i},\mu_{\hat{y_{i}}})}$ (17)

Where$\gamma$is a scaling parameter ensuring the score falls within the range [0,1]; a smaller distance yields a higher score.

1. Based on the complete adjacency matrix A, we compute the average weight of edges connecting the current cell to others within the same cluster. A higher average weight indicates stronger structural consistency. The Neighborhood Consistency Score is defined as:

$S_{i}^{nei}=\frac{1}{{|S}_{\hat{y_{i}}}|-1}\sum_{j\epsilon S_{\hat{y_{i}}, j\neq1}} A_{ij}$ (18)

1. The two scores are then integrated:

$score_{i}=\theta*S_{i}^{dist}+(1-\theta)S_{i}^{nei}$ (19)

Where θ is a weighting coefficient that balances the contribution of the centroid distance score and the neighborhood consistency score.

1. High-confidence samples are selected as $x_{\mathrm{real}}=\left\{ x_{1,},x_{2},...,x_{i} \right\}$, representing the subset of cells identified as high-confidence representatives from each cluster.

**REFERENCES**

1. Horowitz, A., et al., *C1QA is an invariant biomarker for tissue macrophages.* bioRxiv, 2024.

2. Ji, K., et al., *SIRPα blockade improves the antitumor immunity of radiotherapy in colorectal cancer.* Nature, 2023. **9**(1): p. 180.

3. Schupp, J.C., et al., *Integrated Single-Cell Atlas of Endothelial Cells of the Human Lung.* Circulation, 2021. **144**(4): p. 286-302.

4. Rudat, C., et al., *Upk3b is dispensable for development and integrity of urothelium and mesothelium.* PLoS One, 2014. **9**(11): p. e112112.
